# Supplementary material for: GBA1 in Parkinson’s disease: variant detection and pathogenicity scoring matters
Source: BMC Genomics. 2023 Jun 13;24:322. doi: 10.1186/s12864-023-09417-y (PMC10262508; doi:10.1186/s12864-023-09417-y)
Supplement: Supplementary file 1 — Supplementary Material 1 [file 12864_2023_9417_MOESM1_ESM.pdf]

**Supplementary Information:**

# *GBA1* in Parkinson's disease: variant detection and pathogenicity scoring matters

Carolin Gabbert[1], MSc, Susen Schaake[1], BSc, Theresa Lüth[1], MSc, Christoph Much[1], Christine Klein[1], MD, Jan O. Aasly[2], MD, Matthew J. Farrer[3], PhD, Joanne Trinh[1*], PhD

[1]Institute of Neurogenetics, University of Lübeck, Lübeck, Germany

[2]Department of Neuromedicine and Movement Science, Norwegian University of Science and Technology, Trondheim, Norway

[3]Department of Neurology, University of Florida, Gainesville, Florida

*** Correspondence:**

Joanne Trinh

University of Lübeck

Ratzeburger Allee 160

23538 Lübeck, Germany

Email: joanne.trinh@neuro.uni-luebeck.de

Tel.: +49-451-31018202

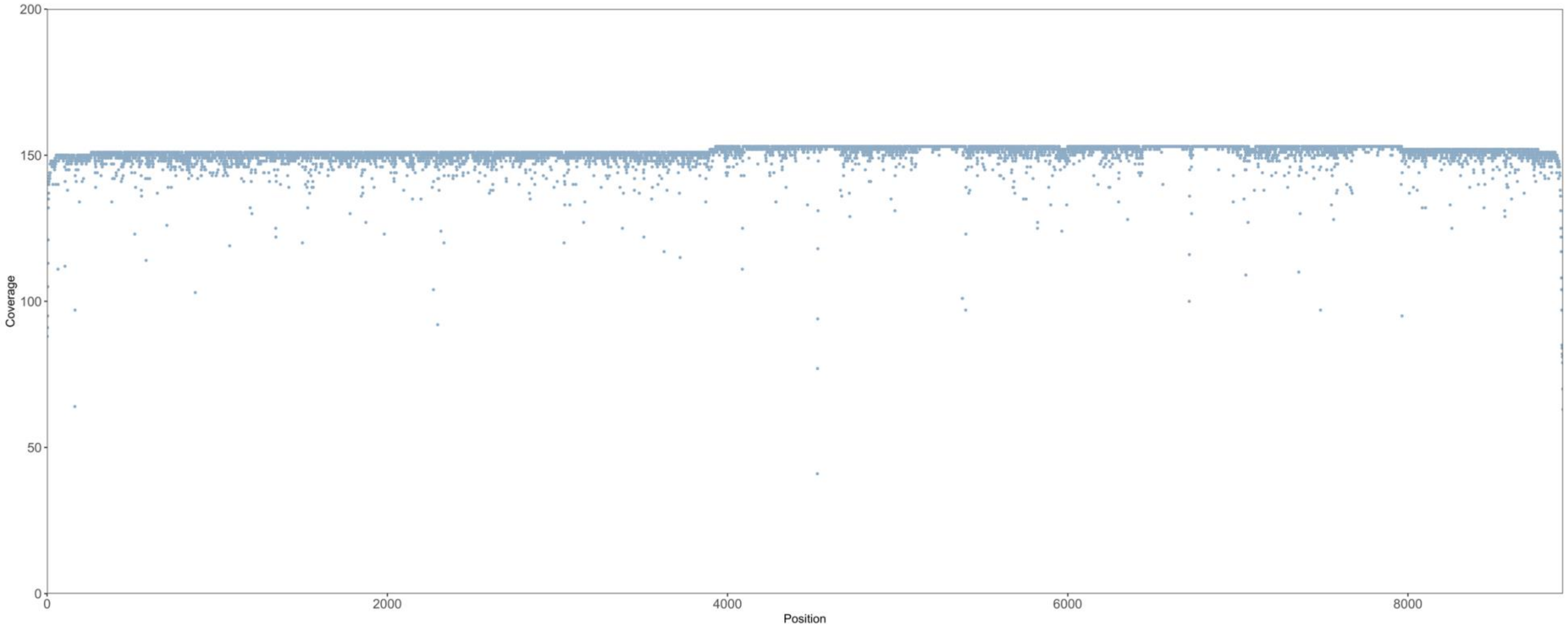


**Supplementary Figure 1.** Exemplary coverage plot. The coverage is shown over the full *GBA1* amplicon (8.9 kb)

**Supplementary Table 1.** Overview over all rare *GBA1* variants detected with Nanopore sequencing

| Position chr1 (hg38) | REF | ALT | Region | SNP ID | Variant information *GBA* (NM_000157.4) | AA change | ACMG classification | ClinVar | SIFT | Poly-phen2 HDIV | Poly-phen2 HVAR | CADD raw | CADD phred | GERP++ | gnomAD (All) |
|---|---|---|---|---|---|---|---|---|---|---|---|---|---|---|---|
| 155232724 | C | T | intergenic | rs578095239 | . | . | Likely benign<br>-1 points = 1 P - 2 B | . | . | . | . | . | . | . | 0.0002 |
| 155232838 | C | T | intergenic | rs561692357 | . | . | Uncertain significance<br>1 points = 1 P - 0 B | . | . | . | . | . | . | . | . |
| 155232871 | T | C | intergenic | rs529155642 | . | . | Likely benign<br>-1 points = 1 P - 2 B | . | . | . | . | . | . | . | 0.0006 |
| 155232982 | G | A | intergenic | rs530421143 | . | . | Likely benign<br>-1 points = 1 P - 2 B | . | . | . | . | . | . | . | 0.001 |
| 155233134 | C | T | intergenic | rs557608543 | . | . | Uncertain significance<br>0 points = 1 P - 1 B | . | . | . | . | . | . | . | 0.0005 |
| 155233268 | C | G | intergenic | rs367634752 | . | . | Likely benign<br>-1 points = 1 P - 2 B | . | . | . | . | . | . | . | 0.0016 |
| 155233286 | C | T | intergenic | rs1042594015 | . | . | Likely benign<br>-1 points = 1 P - 2 B | . | . | . | . | . | . | . | . |
| 155233287 | G | A | intergenic | rs368998334 | . | . | Likely benign<br>-6 points = 0 P - 6 B | . | . | . | . | . | . | . | 0.0032 |
| 155233514 | A | G | downstream | rs2361530 | . | . | Likely benign<br>-1 points = 1 P - 2 B | . | . | . | . | . | . | . | . |
| 155233517 | C | T | downstream | rs2361531 | . | . | Likely benign<br>-1 points = 1 P - 2 B | . | . | . | . | . | . | . | . |
| 155233521 | T | A | downstream | rs2361532 | . | . | Likely benign<br>-1 points = 1 P - 2 B | . | . | . | . | . | . | . | . |
| 155233531 | G | A | downstream | rs2361533 | . | . | Uncertain significance<br>0 points = 1 P - 1 B | . | . | . | . | . | . | . | . |
| 155233541 | G | T | downstream | rs4024047 | . | . | Likely benign<br>-1 points = 1 P - 2 B | . | . | . | . | . | . | . | 0.00003238 |
| 155233543 | G | A | downstream | rs4024048 | . | . | Likely benign<br>-1 points = 1 P - 2 B | . | . | . | . | . | . | . | 0.00003241 |
| 155233549 | C | T | downstream | rs4024049 | . | . | Likely benign<br>-1 points = 1 P - 2 B | . | . | . | . | . | . | . | 0.00003258 |
| 155233612 | A | G | downstream | rs2142046 | . | . | Benign<br>-10 points = 0 P - 10 B | . | . | . | . | . | . | . | 0.0024 |
| 155233639 | G | A | downstream | rs2142045 | . | . | Likely benign<br>-1 points = 1 P - 2 B | . | . | . | . | . | . | . | 0.0005 |
| 155234414 | G | A | downstream | rs201209118 | . | . | Benign<br>-10 points = 0 P - 10 B | . | . | . | . | . | . | . | 0 |
| 155234893 | A | G | UTR3 | rs368275143 | c.*102T>C | . | Likely benign<br>-1 points = 1 P - 2 B | . | . | . | . | . | . | . | 0.0113 |
| 155234903 | C | T | UTR3 | rs708606 | c.*92G>A | . | Likely benign<br>-1 points = 1 P - 2 B | . | . | . | . | . | . | . | 0.0022 |
| 155235104 | G | A | intronic | rs374690110 | c.1506-4C>T | . | Likely benign<br>-1 points = 1 P - 2 B | . | . | . | . | . | . | . | 0.0002 |
| 155235206 | G | A | exonic | rs371779859 | c.1494C>T | p.Val498= | Likely benign<br>-2 points = 1 P - 3 B | . | . | . | . | . | . | . | 0.00009689 |
| 155235222 | C | T | exonic | . | c.1478G>A | p.Gly493Asp | Uncertain significance<br>1 points = 1 P - 0 B | . | T | D | P | 4.834 | 24.8 | 3.16 | . |
| 155235252 | A | G | exonic | rs421016 | c.1448T>C | p.Leu483Pro | Pathogenic<br>11 points = 11 P – 0 B | Pathogenic; risk factor | D | P | P | 4.842 | 24.8 | 3.16 | 0.0007 |
| 155235302 | A | G | exonic | . | c.1398T>C | p.Ile466= | Likely benign<br>-2 points = 1 P - 3 B | . | . | . | . | . | . | . | . |
| 155235344 | G | A | intronic | rs569191841 | c.1389-33C>T | . | Likely benign<br>-1 points = 1 P - 2 B | . | . | . | . | . | . | . | 0.00003234 |

| | | | | | | | | | | | | | | | |
|---|---|---|---|---|---|---|---|---|---|---|---|---|---|---|---|
| 155235379 | A | G | intronic | rs2974924 | c.1389-68T>C | . | Likely benign<br>-1 points = 1 P - 2 B | . | . | . | . | . | . | . | 0.0105 |
| 155235587 | C | T | intronic | rs12752133 | c.1388+94G>A | . | Likely benign<br>-1 points = 1 P - 2 B | . | . | . | . | . | . | . | 0.0127 |
| 155235791 | G | A | exonic | rs201499639 | c.1278C>T | p.Pro426= | Likely benign<br>-2 points = 1 P - 3 B | Likely benign | . | . | . | . | . | . | 0.0000323 |
| 155235843 | T | C | exonic | rs76763715 | c.1226A>G | p.Asn409Ser | Uncertain significance<br>5 points = 5 P – 0 B | Pathogenic/Likely pathogenic; risk factor | D | P | B | 3.202 | 22.7 | 3.53 | 0.0017 |
| 155235928 | G | C | intronic | rs41264925 | c.1225-84C>G | . | Likely benign<br>-1 points = 1 P - 2 B | . | . | . | . | . | . | . | . |
| 155236050 | G | A | intronic | rs1036605613 | c.1224+195C>T | . | Uncertain significance<br>0 points = 1 P – 1 B | . | . | . | . | . | . | . | 0.00003232 |
| 155236246 | G | A | exonic | rs75548401 | c.1223C>T | p.Thr408Met | Uncertain significance<br>1 points = 1 P – 0 B | Uncertain significance (2); Benign (4); Likely benign (3) | T | B | B | 2.993 | 22.2 | 3.57 | 0.0076 |
| 155236294 | C | T | exonic | rs11558184 | c.1175G>A | p.Arg392Gln | Uncertain significance<br>1 points = 1 P – 0 B | . | T | P | B | 3.323 | 22.9 | 3.67 | 0.00003233 |
| 155236331 | C | T | exonic | rs781306264 | c.1138G>A | p.Ala380Thr | Uncertain significance<br>1 points = 1 P – 0 B | . | D | D | D | 6.843 | 33 | 3.67 | . |
| 155236366 | C | T | exonic | rs1064648 | c.1103G>A | p.Arg368His | Uncertain significance<br>1 points = 1 P – 0 B | . | T | B | B | 3.16 | 22.6 | 2.75 | . |
| 155236367 | G | A | exonic | rs374306700 | c.1102C>T | p.Arg368Cys | Uncertain significance<br>1 points = 1 P – 0 B | Likely pathogenic (2); Uncertain significance (2) | T | D | P | 6.006 | 27.9 | 3.67 | . |
| 155236376 | C | T | exonic | rs2230288 | c.1093G>A | p.Glu365Lys | Likely benign<br>-1 points = 1 P – 2 B | Benign/Likely benign; risk factor | T | B | B | 2.173 | 17.33 | 3.67 | 0.0128 |
| 155236459 | T | C | exonic | rs1306645655 | c.1010A>G | p.Asp337Gly | Uncertain significance<br>1 points = 1 P – 0 B | . | D | D | D | 5.661 | 26.7 | 3.67 | . |
| 155236558 | C | T | intronic | rs772645370 | c.1000-89G>A | . | Likely benign<br>-1 points = 1 P – 2 B | . | . | . | . | . | . | . | 0.0002 |
| 155236787 | G | A | intronic | rs531447697 | c.1000-318C>T | . | Likely benign<br>-1 points = 1 P – 2 B | . | . | . | . | . | . | . | 0.00003263 |
| 155237162 | G | A | intronic | rs547873878 | c.999+179C>T | . | Likely benign<br>-1 points = 1 P – 2 B | . | . | . | . | . | . | . | 0.0003 |
| 155237222 | A | C | intronic | rs946743963 | c.999+119T>G | . | Likely benign<br>-1 points = 1 P – 2 B | . | . | . | . | . | . | . | 0.00003232 |
| 155237239 | G | A | intronic | rs72704130 | c.999+102C>T | . | Likely benign<br>-1 points = 1 P – 2 B | . | . | . | . | . | . | . | 0.0138 |
| 155237265 | G | A | intronic | rs556277010 | c.999+76C>T | . | Likely benign<br>-1 points = 1 P – 2 B | . | . | . | . | . | . | . | 0.00003232 |
| 155237412 | T | C | exonic | rs1057942 | c.928A>G | p.Ser310Gly | Uncertain significance<br>3 points = 3 P – 0 B | Pathogenic/Likely pathogenic | T | P | B | 1.77 | 14.81 | 3.51 | 0.00006461 |
| 155237438 | C | T | exonic | rs140955685 | c.902G>A | p.Arg301His | Uncertain significance<br>1 points = 1 P – 0 B | Uncertain significance | T | B | B | 2.204 | 17.54 | 2.59 | 0.0003 |
| 155237596 | A | T | intronic | rs140335079 | c.762-18T>G | . | Likely benign<br>-1 points = 1 P – 2 B | . | . | . | . | . | . | . | 0.009 |
| 155237623 | C | G | intronic | rs377217353 | c.762-45G>C | . | Likely benign<br>-1 points = 1 P – 2 B | . | . | . | . | . | . | . | 0.00003236 |
| 155237914 | A | G | intronic | rs549565365 | c.761+220T>C | . | Likely benign<br>-1 points = 1 P – 2 B | . | . | . | . | . | . | . | 0.0045 |
| 155238057 | G | T | intronic | rs183540501 | c.761+77C>A | . | Likely benign<br>-1 points = 1 P – 2 B | . | . | . | . | . | . | . | 0.0031 |
| 155238175 | G | A | exonic | rs376613535 | c.720C>T | p.Pro240= | Likely benign<br>-2 points = 1 P – 3 B | . | . | . | . | . | . | . | 0.00006465 |
| 155238265 | G | A | exonic | rs201615998 | c.630C>T | p.Pro210= | Likely benign<br>-2 points = 1 P – 3 B | . | . | . | . | . | . | . | . |

| | | | | | | | | | | | | | | | |
|---|---|---|---|---|---|---|---|---|---|---|---|---|---|---|---|
| 155238570 | C | G | exonic | rs147138516 | c.535G>C | p.Asp179His | Uncertain significance<br>1 points = 1 P – 0 B | Likely pathogenic (1); Uncertain significance (2) | T | P | P | 0.611 | 8.229 | 2.62 | 0.00009723 |
| 155238629 | C | T | exonic | rs79653797 | c.476G>A | p.Arg159Gln | Uncertain significance<br>3 points = 3 P – 0 B | Pathogenic/Likely pathogenic | D | D | D | 6.336 | 29.3 | 3.55 | . |
| 155238630 | G | A | exonic | rs439898 | c.475C>T | p.Arg159Trp | Uncertain significance<br>3 points = 3 P – 0 B | Pathogenic | D | D | D | 6.19 | 28.6 | 3.55 | 0.00003238 |
| 155238631 | G | A | exonic | rs147411159 | c.474C>T | p.Ile158= | Likely benign<br>-2 points = 1 P – 3 B | Uncertain significance (1); Likely benign (3) | . | . | . | . | . | . | 0.0006 |
| 155238833 | A | G | intronic | rs188328778 | c.455-183T>C | . | Likely benign<br>-1 points = 1 P – 2 B | . | . | . | . | . | . | . | 0.0119 |
| 155238857 | G | A | intronic | rs1042674060 | c.455-207C>T | . | Likely benign<br>-1 points = 1 P – 2 B | . | . | . | . | . | . | . | 0.0001 |
| 155238927 | A | G | intronic | rs778649863 | c.455-277T>C | . | Likely benign<br>-1 points = 1 P – 2 B | . | . | . | . | . | . | . | . |
| 155238984 | C | G | intronic | rs752258174 | c.455-334G>C | . | Likely benign<br>-1 points = 1 P – 2 B | . | . | . | . | . | . | . | . |
| 155238985 | G | A | intronic | rs951266434 | c.455-335C>T | . | Likely benign<br>-1 points = 1 P – 2 B | . | . | . | . | . | . | . | . |
| 155239079 | G | A | intronic | rs572108051 | c.455-429C>T | . | Likely benign<br>-1 points = 1 P – 2 B | . | . | . | . | . | . | . | 0 |
| 155239287 | C | T | intronic | rs1005434278 | c.454+329G>A | . | Likely benign<br>-1 points = 1 P – 2 B | . | . | . | . | . | . | . | 0.00003262 |
| 155239509 | C | T | intronic | rs570088632 | c.454+107G>A | . | Likely benign<br>-1 points = 1 P – 2 B | . | . | . | . | . | . | . | . |
| 155239633 | G | T | exonic | rs758447515 | c.437C>A | p.Ser146Ter | Likely pathogenic<br>9 points = 9 P – 0 B | . | . | . | . | 10.665 | 36 | 3.25 | . |
| 155239858 | C | A | intronic | rs369792423 | c.307+28G>T | . | Likely benign<br>-1 points = 1 P – 2 B | . | . | . | . | . | . | . | 0.00006462 |
| 155239939 | C | T | exonic | rs77829017 | c.254G>A | p.Gly85Glu | Uncertain significance<br>1 points = 1 P – 0 B | Pathogenic | D | D | D | 4.841 | 24.8 | 3.46 | . |
| 155239961 | G | A | exonic | rs146774384 | c.232C>T | p.Arg78Cys | Uncertain significance<br>1 points = 1 P – 0 B | . | T | D | P | 4.822 | 24.8 | 3.46 | 0.00009697 |
| 155240072 | G | C | exonic | . | c.121C>G | p.Arg41Gly | Uncertain significance<br>1 points = 1 P – 0 B | . | T | B | B | 0.931 | 10.26 | 3.41 | . |
| 155240122 | T | G | intronic | rs199565854 | c.116-45A>C | . | Likely benign<br>-1 points = 1 P – 2 B | . | . | . | . | . | . | . | 0.0003 |
| 155240171 | C | T | intronic | rs114217696 | c.116-94G>A | . | Likely benign<br>-1 points = 1 P – 2 B | . | . | . | . | . | . | . | 0.008 |
| 155240336 | C | T | intronic | rs142348200 | c.116-259G>A | . | Likely benign<br>-1 points = 1 P – 2 B | . | . | . | . | . | . | . | 0.0012 |
| 155240779 | T | C | intronic | rs2361534 | c.28-62A>G | . | Likely benign<br>-1 points = 1 P – 2 B | . | . | . | . | . | . | . | 0.001 |
| 155240816 | G | A | intronic | rs940168433 | c.28-99C>T | . | Likely benign<br>-1 points = 1 P – 2 B | . | . | . | . | . | . | . | . |
| 155241114 | C | T | UTR5 | rs1141801 | c.-2G>T | . | Likely benign<br>-1 points = 1 P – 2 B | . | . | . | . | . | . | . | . |
| 155241127 | T | C | UTR5 | rs41264927 | c.-15A>G | . | Likely benign<br>-1 points = 1 P – 2 B | . | . | . | . | . | . | . | 0.0012 |
| 155241257 | C | T | intronic | rs371157845 | NM_001005742.3:c.-49-96G>A | . | Likely benign<br>-1 points = 1 P – 2 B | . | . | . | . | . | . | . | . |
| 155241315 | T | C | intronic | rs188978150 | NM_001005742.3:c.-49-154A>G | . | Likely benign<br>-1 points = 1 P – 2 B | Uncertain Significance | . | . | . | . | . | . | 0.0086 |

Variants with a gnomAD frequency >2% and without information on SNP ID or amino acid change were excluded. Pathogenicity scores were used from ACMG, Varsome, Clinvar, SIFT, Polphen2, CADD, and GERP++. Variants that were Sanger sequenced are highlighted in gray.

**Supplementary Table 2.** Rare *GBA1* variants (predicted as "pathogenic"/"likely pathogenic"/"uncertain significance") sequenced with the Oxford Nanopore and confirmed with Sanger sequencing

| ***GBA1* variant** | **cDNA transcript GBA(NM_000157.4)** | **Exon number** | **PD cases (n=462)** | **Controls (n=367)** |
|---|---|---|---|---|
| p.R78C | c.232C>T | 3 | 2 | 0 |
| p.S146X | c.437C>A | 4 | 1 | 0 |
| p.R159W | c.475C>T | 5 | 12 | 1 |
| p.R301H | c.902G>A | 7 | 0 | 1 |
| p.S310G | c.928A>G | 7 | 1 | 1 |
| p.D337G | c.1010A>G | 8 | 2 | 1 |
| p.E365K | c.1093G>A | 8 | 33 | 10 |
| p.R368C | c.1102C>T | 8 | 2 | 0 |
| p.A380T | c.1138G>A | 8 | 1 | 0 |
| p.T408M | c.1223C>T | 8 | 10 | 13 |
| p.N409S | c.1226A>G | 9 | 13 | 4 |
| p.L483P | c.1448T>C | 10 | 6 | 0 |
| p.G493D | c.1478G>A | 10 | 1 | 0 |

**Supplementary Table 3.** Publications on *GBA1* variants and frequencies in patients with PD included in PubMed

| Authors, Year (PMID) | n (PD/control) | Population/ Region | Method | *GBA1* variants screened* | *GBA1* variants found* | n mutation carriers/n total (frequency)* |
|---|---|---|---|---|---|---|
| Toft et al., 2006 (1) (16476943) | 311/474 | Norwegian | Variant screening | L444P, N370S | L444P, N370S | L444P: PD: 3/311 (0.96%), Controls: 1/474 (0.21%)<br>N370S: PD: 4/311 (1.29%), Controls: 7/474 (1.48%) |
| Lunde et al., 2018 (2) (29792872) | 442/419 | Norwegian | Genotyping | N370S, T369M, E326K, V460L, Y135C, L444P | N370S, T369M, E326K, V460L, Y135C, L444P | All: PD: 53/442 (12.0%), Controls: 29/419 (6.9%)<br>N370S: PD: 1/442 (0.2%), Controls: 1/419 (0.2%)<br>T369M: PD: 7/442 (1.7%), Controls: 16/419 (3.6%)<br>E326K: PD: 18/442 (4.3%), Controls: 29/419 (6.6%)<br>V460L: PD: 1/442 (0.2%), Controls: 1/419 (0.2%)<br>Y135C: PD: 0/442 (0%), Controls: 1/419 (0.2%)<br>L444P: PD: 2/442 (0.5%), Controls: 6/419 (1.4%) |
| Berge-Seidl et al., 2017 (3) (28830825) | 1152/713 | Scandinavian | Targeted deep sequencing; genotyping | All *GBA1* exons | E326K, T369M, N370S, R463C, IVS3+1G>A, V457A, G377D, W357R | E326K: PD: 20/330 (6.06%)<br>T369M: PD: 13/366 (3.55%)<br>N370S: PD: 1/339 (0.29%)<br>R463C: PD: 1/366 (0.27%)<br>IVS3+1G>A: PD: 1/366 (0.27%)<br>V457A: PD: 1/366 (0.27%)<br>G377D: PD: 1/366 (0.27%)<br>W357R: PD: 1/366 (0.27%) |
| Ran et al., 2022 (4)] (35779693) | 1131/1594 | Swedish | Pyrosequencing for genotyping of T369M | T369M | T369M | T369M: PD: 47/1091 (4.31%), Controls: 50/1474 (3.39%) |
| Ran et al., 2016 (5)] (27255555) | 1625/2025 | Swedish | Genotyping by pyrosequencing of E326K, N370S, and L444P | E326K, N370S, L444P | E326K, N370S, L444P | E326K: PD: 90/1625 (5.54%), Controls: 65/2025 (3.21%)<br>N370S: PD: 10/1625 (0.62%), Controls: 2/2025 (0.10%)<br>L444P: PD: 35/1625 (2.15%), Controls: 65/2025 (0.15%) |
| Ylönen et al., 2017 (6) (29029963) | 852/403 | Finnish | Variant screening for N370S and L444P; Whole exome sequencing in 225 EOPD cases | All *GBA1* exons; N370S, L444P | N370S, L444P | N370S: PD: 4/852 (0.5%), Controls: 1/403 (0.2%)<br>L444P: PD: 17/852 (2.0%), Controls: 2/403 (0.5%) |
| Muldmaa et al., 2021 (7) (32740907) | 189/158 | Estonian | Next-generation sequencing | NA | L444P, T369M, E326K, L276I, E10X | All: PD: 19/189 (10.1%), Controls: 6/158 (3.8%)<br>*GBA1*-related risk variants: 18/189 (9.5%)<br>L444P: PD: 1/189 (0.5%), Controls: 0/158 (0%)<br>T369M: PD: 10/189 (5.3%), Controls: 3/158 (1.9%)<br>E326K: PD: 6/189 (3.2%), Controls: 3/158 (1.9%)<br>L276I: PD: 1/189 (0.5%), Controls: 0/158 (0%)<br>E10X: PD: 1/189 (0.5%), Controls: 0/158 (0%) |
| Neumann et al., 2009 (8) (19286695) | 790/257 | British | DNA sequencing of full *GBA1* gene | All *GBA1* exons and the flanking introns | L483P, D482N, R502C, RecNciI (L483P, A495P, V499V), RecA456P (L483P, A495P), N409S, D448H, D419A, N421PfsX4, R296Q, G232E, R170C, K46E, V497L | All: PD: 33/790 (4.18%), Controls: 3/257 (1.17%)<br>L483P: PD: 11/790 (1.39%), Controls: 0/257 (0%)<br>D482N: PD: 1/790 (0.13%), Controls: 0/257 (0%)<br>R502C: PD: 3/790 (0.38%), Controls: 0/257 (0%)<br>RecNciI (L483P, A495P, V499V): PD: 2/790 (0.25%), Controls: 0/257 (0%)<br>RecA456P (L483P, A495P): PD: 1/790 (0.13%), Controls: 0/257 (0%)<br>N409S: PD: 8/790 (1.01%), Controls: 1/257 (0.39%)<br>D448H: PD: 1/790 (0.13%), Controls: 0/257 (0%)<br>D419A: PD: 1/790 (0.13%), Controls: 0/257 (0%)<br>N421PfsX4: PD: 1/790 (0.13%), Controls: 0/257 (0%)<br>R296Q: PD: 1/790 (0.13%), Controls: 1/257 (0.39%)<br>G232E: PD: 1/790 (0.13%), Controls: 0/257 (0%)<br>R170C: PD: 1/790 (0.13%), Controls: 0/257 (0%)<br>K46E: PD: 1/790 (0.13%), Controls: 0/257 (0%)<br>V497L: PD: 0/790 (0%), Controls: 1/257 (0.39%) |

| | | | | | | |
|---|---|---|---|---|---|---|
| Winder-Rhodes et al., 2013 (9) (23413260) | 259/0 | British (2 South Asian) | DNA sequencing of all *GBA1* exons | All *GBA1* exons | L444P, N370S, N462K, R463C, R257Q, E326K, T369M, E388K, L119L | All: PD: 9/259 (3.5%)<br>L444P: PD: 3/259 (1.2%)<br>N370S: PD: 3/259 (1.2%)<br>N462K: PD: 1/259 (0.4%)<br>R463C: PD: 1/259 (0.4%)<br>R257Q: PD: 1/259 (0.4%)<br>E326K: PD: 8/259 (3.1%)<br>T369M: PD: 5/259 (1.9%)<br>E388K: PD: 1/259 (0.4%)<br>L119L: PD: 1/259 (0.4%) |
| Duran et al., 2013 (10) (23225227) | 185/283 | UK Caucasian | Sanger sequencing of the full *GBA1* gene | Full *GBA1* gene | N370S, L444P, RecNciI (L444P+A456P+V460V), R463C, E326K, IVS2+1, R131C, W184R, N188S, H255Q, R257Q, D409H, RecTL, E388K, G113A, T369M, S465P, L(−14)V, V172L, S177T, L217P, L317L, L354P, V375G, IVS10-4 C>T, IVS10-12 C>T, E340A, V458L | All: PD: 48/185 (25.94%), Controls: 12/283 (4.24%)<br>All pathogenic: PD: 37/185 (20%), Controls: 9/283 (3.18%)<br>N370S: PD: 5/185 (2.70%), Controls: 1/283 (0.35%)<br>L444P: PD: 2/185 (1.08%), Controls: 0/283 (0%)<br>RecNciI (L444P+A456P+V460V): PD: 3/185 (1.62%), Controls: 0/283 (0%)<br>R463C: PD: 3/185 (1.62%), Controls: 0/283 (0%)<br>E326K: PD: 14/185 (7.57%), Controls: 7/283 (2.47%), 6/202 (2.97%)<br>IVS2+1: PD: 1/185 (0.54%), Controls: 0/283 (0%)<br>R131C: PD: 2/185 (1.08%), Controls: 0/283 (0%)<br>W184R: PD: 1/185 (0.54%), Controls: 0/283 (0%)<br>N188S: PD: 1/185 (0.54%), Controls: 0/283 (0%)<br>H255Q: PD: 1/185 (0.54%), Controls: 0/283 (0%)<br>R257Q: PD: 1/185 (0.54%), Controls: 1/283 (0.35%)<br>D409H: PD: 2/185 (1.08%), Controls: 0/283 (0%)<br>RecTL: PD: 1/185 (0.54%), Controls: 0/283 (0%)<br>E388K: PD: 1/185 (0.54%), Controls: 0/283 (0%)<br>G113A: PD: 2/185 (1.08%), Controls: 0/283 (0%)<br>T369M: PD: 1/185 (0.54%), Controls: 1/283 (0.35%)<br>S465P: PD: 1/185 (0.54%), Controls: 0/283 (0%)<br>L(−14)V: PD: 1/185 (0.54%), Controls: 0/283 (0%)<br>V172L: PD: 2/185 (1.08%), Controls: 0/283 (0%)<br>S177T: PD: 1/185 (0.54%), Controls: 0/283 (0%)<br>L217P: PD: 1/185 (0.54%), Controls: 0/283 (0%)<br>L317L: PD: 1/185 (0.54%), Controls: 0/283 (0%)<br>L354P: PD: 1/185 (0.54%), Controls: 0/283 (0%)<br>V375G: PD: 1/185 (0.54%), Controls: 0/283 (0%)<br>IVS10-4 C>T: PD: 1/185 (0.54%), Controls: 0/283 (0%)<br>IVS10-12 C>T: PD: 1/185 (0.54%), Controls: 0/283 (0%)<br>E340A: PD: 0/185 (0%), Controls: 1/283 (0.35%)<br>V458L: PD: 0/185 (0%), Controls: 1/283 (0.35%) |
| Olszewska et al., 2020 (11) (32714263) | 314/96 (friends or spouses) | Irish | DNA sequencing of all *GBA1* exons | All *GBA1* exons | T408M, E365K, F255Y, N409S, D448H, L483P, A495P, V499V, G416C, G234E, R301H, R368C | T408M: PD: 6/314 (1.91%), Controls: 4/96 (4.17%)<br>E365K: PD: 13/314 (4.14%), Controls: 4/96 (4.17%)<br>F255Y: PD: 1/314 (0.32%), Controls: 0/96 (0%)<br>N409S: PD: 3/314 (0.96%), Controls: 0/96 (0%)<br>D448H: PD: 1/314 (0.32%), Controls: 0/96 (0%)<br>L483P: PD: 3/314 (0.96%), Controls: 0/96 (0%)<br>A495P: PD: 3/314 (0.96%), Controls: 0/96 (0%)<br>V499V: PD: 3/314 (0.96%), Controls: 0/96 (0%)<br>G416C: PD: 1/314 (0.32%), Controls: 0/96 (0%)<br>G234E: PD: 1/314 (0.32%), Controls: 0/96 (0%)<br>R301H: PD: 1/314 (0.32%), Controls: 0/96 (0%)<br>R368C: PD: 1/314 (0.32%), Controls: 0/96 (0%) |

| | | | | | | |
|---|---|---|---|---|---|---|
| Crosiers et al., 2016 (12) (27397011) | 266/536 | Flanders-Belgian | In-depth Sanger sequencing of all *GBA1* exons | All *GBA1* exons | D179H, Q256SfsX9, L363P, N409S, L483P, RecNcil (L483P-A495S-V499V), E365K, T408M, G39R, H529R | All rare: PD: 12/266 (4.5%), Controls: 2/536 (0.37%)<br>D179H: PD: 1/266 (0.4%), Controls: 0/536 (0%)<br>Q256SfsX9: PD: 1/266 (0.4%), Controls: 0/536 (0%)<br>L363P: PD: 1/266 (0.4%), Controls: 0/536 (0%)<br>N409S: PD: 3/266 (1.1%), Controls: 1/536 (0.2%)<br>L483P: PD: 3/266 (1.1%), Controls: 1/536 (0.2%)<br>RecNcil (L483P-A495S-V499V): PD: 1/266 (0.4%), Controls: 0/536 (0%)<br>E365K: PD: 12/266 (4.5%), Controls: 15/536 (2.8%)<br>T408M: PD: 3/266 (1.1%), Controls: 11/536 (2.0%)<br>G39R: PD: 1/266 (0.4%), Controls: 0/536 (0%)<br>H529R: PD: 1/266 (0.4%), Controls: 0/536 (0%) |
| den Heijer et al., 2020 (13) (32618053) | 3402/655 | Dutch | Next-generation sequencing of full *GBA1* gene | Full *GBA1* gene | E-30Gfs*8, L-24S, L-24S+S23G, Q-7R, C18*, R39C, S45Rfs*15, R120W, D140H, R170H, A190T, G202R, F216Y, G250S, H255Q, I260T, L324P, G325R, E326K, R329C, W348G, Q350H, T369M, N370S, V375G, D380Y, E388K, N392S, D409H, L444P, D453L, V460M, R463P, S484L, S488T, H490R, L268=, S271G, A456P, V460=, S-1T, V459=, R496H, G390E, V17=, T61=, I119=, I130=, Q143=, G193=, G195=, G344=, T369=, P452=, c.762-5G>A, c.1000-4G>T | E-30Gfs*8: PD: 1/3402 (0.03%), Controls: 0/655 (0%)<br>L-24S: PD: 1/3402 (0.03%), Controls: 0/655 (0%)<br>L-24S+S23G: PD: 1/3402 (0.03%), Controls: 0/655 (0%)<br>Q-7R: PD: 2/3402 (0.06%), Controls: 0/655 (0%)<br>C18*: PD: 1/3402 (0.03%), Controls: 0/655 (0%)<br>R39C: PD: 1/3402 (0.03%), Controls: 0/655 (0%)<br>S45Rfs*15: PD: 1/3402 (0.03%), Controls: 0/655 (0%)<br>R120W: PD: 5/3402 (0.15%), Controls: 0/655 (0%)<br>D140H: PD: 84/3402 (2.47%), Controls: 6/655 (0.92%)<br>R170H: PD: 2/3402 (0.06%), Controls: 0/655 (0%)<br>A190T: PD: 1/3402 (0.03%), Controls: 0/655 (0%)<br>G202R: PD: 1/3402 (0.03%), Controls: 0/655 (0%)<br>F216Y: PD: 1/3402 (0.03%), Controls: 0/655 (0%)<br>G250S: PD: 1/3402 (0.03%), Controls: 0/655 (0%)<br>H255Q: PD: 2/3402 (0.06%), Controls: 0/655 (0%)<br>I260T: PD: 2/3402 (0.06%), Controls: 0/655 (0%)<br>L324P: PD: 2/3402 (0.06%), Controls: 1/655 (0.15%)<br>G325R: PD: 1/3402 (0.03%), Controls: 0/655 (0%)<br>E326K: PD: 314/3402 (9.23%), Controls: 18/655 (2.75%)<br>R329C: PD: 2/3402 (0.06%), Controls: 0/655 (0%)<br>W348G: PD: 1/3402 (0.03%), Controls: 0/655 (0%)<br>Q350H: PD: 1/3402 (0.03%), Controls: 1/655 (0.15%)<br>T369M: PD: 98/3402 (2.88%), Controls: 12/655 (1.83%)<br>N370S: PD: 32/3402 (0.94%), Controls: 2/655 (0.31%)<br>V375G: PD: 1/3402 (0.03%), Controls: 0/655 (0%)<br>D380Y: PD: 1/3402 (0.03%), Controls: 0/655 (0%)<br>E388K: PD: 3/3402 (0.09%), Controls: 0/655 (0%)<br>N392S: PD: 1/3402 (0.03%), Controls: 0/655 (0%)<br>D409H: PD: 1/3402 (0.03%), Controls: 0/655 (0%)<br>L444P: PD: 26/3402 (0.76%), Controls: 0/655 (0%)<br>D453L: PD: 5/3402 (0.15%), Controls: 0/655 (0%)<br>V460M: PD: 1/3402 (0.03%), Controls: 0/655 (0%)<br>R463P: PD: 2/3402 (0.06%), Controls: 1/655 (0.15%)<br>S484L: PD: 1/3402 (0.03%), Controls: 0/655 (0%)<br>S488T: PD: 1/3402 (0.03%), Controls: 0/655 (0%)<br>H490R: PD: 1/3402 (0.03%), Controls: 0/655 (0%)<br>L268=+S271G+D409H: PD: 1/3402 (0.03%), Controls: 0/655 (0%)<br>RecTL (D409H+L444P+A456P+V460=): PD: 1/3402 (0.03%), Controls: 0/655 (0%)<br>RecNciI (L444P+A456P+V460=): PD: 4/3402 (0.12%), Controls: 0/655 (0%)<br>S-1T: PD: 1/3402 (0.03%), Controls: 0/655 (0%)<br>V459=: PD: 5/3402 (0.15%), Controls: 0/655 (0%)<br>R496H: PD: 1/3402 (0.03%), Controls: 0/655 (0%)<br>G390E: PD: 1/3402 (0.03%), Controls: 1/655 (0.15%)<br>V17=: PD: 0/3402 (0%), Controls: 1/655 (0.15%)<br>T61=: PD: 1/3402 (0.03%), Controls: 0/655 (0%)<br>I119=: PD: 5/3402 (0.15%), Controls: 0/655 (0%) |

| | | | | | | |
|---|---|---|---|---|---|---|
| | | | | | | I130=: PD: 1/3402 (0.03%), Controls: 0/655 (0%)<br>Q143=: PD: 1/3402 (0.03%), Controls: 0/655 (0%)<br>G193=: PD: 1/3402 (0.03%), Controls: 1/655 (0.15%)<br>G195=: PD: 1/3402 (0.03%), Controls: 0/655 (0%)<br>G344=: PD: 1/3402 (0.03%), Controls: 0/655 (0%)<br>T369=: PD: 2/3402 (0.06%), Controls: 0/655 (0%)<br>P452=: PD: 1/3402 (0.03%), Controls: 0/655 (0%)<br>V460=: PD: 6/3402 (0.18%), Controls: 0/655 (0%)<br>c.762-5G>A: PD: 1/3402 (0.03%), Controls: 0/655 (0%)<br>c.1000-4G>T: PD: 0/3402 (0%), Controls: 1/655 (0.15%) |
| Anheim et al., 2012 (14) (22282650) | 525/71 (relatives) | French (88%) | DNA sequencing of all *GBA1* exons | All *GBA1* exons | N370S, L444P, F246L, G202R, R120W, R463C, R463H, S125N, S173SfsX50, S364N, T323I, Y304C, 1263-1217del55bp, E326K | All: PD (probands): 24/525 (4.6%)<br>N370S: PD (probands): 10/525 (1.9%), Relatives with PD: 9/32 (28.1%), Relatives without PD: 12/71 (16.9%)<br>L444P: PD (probands): 5/525 (1.0%), Relatives with PD: 6/32 (18.8%), Relatives without PD: 5/71 (7.0%)<br>F246L: PD (probands): 1/525 (0.2%), Relatives with PD: 0/32 (0%), Relatives without PD: 0/71 (0%)<br>G202R: PD (probands): 1/525 (0.2%), Relatives with PD: 0/32 (0%) Relatives without PD: 0/71 (0%)<br>R120W: PD (probands): 1/525 (0.2%), Relatives with PD: 1/32 (3.1%), Relatives without PD: 0/71 (0%)<br>R463C: PD (probands): 1/525 (0.2%), Relatives with PD: 1/32 (3.1%), Relatives without PD: 1/71 (1.4%)<br>R463H: PD (probands): 1/525 (0.2%), Relatives with PD: 3/32 (9.4%), Relatives without PD: 3/71 (4.2%)<br>S125N: PD (probands): 1/525 (0.2%), Relatives with PD: 1/32 (3.1%), Relatives without PD: 0/71 (0%)<br>S173SfsX50: PD (probands): 1/525 (0.2%), Relatives with PD: 1/32 (3.1%), Relatives without PD: 3/71 (4.2%)<br>S364N: PD (probands): 1/525 (0.2%), Relatives with PD: 1/32 (3.1%), Relatives without PD: 0/71 (0%)<br>T323I: PD (probands): 1/525 (0.2%), Relatives with PD:1/32 (3.1%), Relatives without PD: 1/71 (1.4%)<br>Y304C: PD (probands): 1/525 (0.2%), Relatives with PD: 2/32 (6.3%), Relatives without PD: 6/71 (8.5%)<br>1263-1217del55bp: PD (probands): 1/525 (0.2%), Relatives with PD: 1/32 (3.1%), Relatives without PD: 0/71 (0%)<br>E326K: PD (probands): 0/525 (0%), Relatives with PD: 1/32 (3.1%), Relatives without PD: 0/71 (0%) |
| Lesage et al., 2011 (15) (20947659) | 1130/391 (mainly spouses) | French (89%) | DNA sequencing of full *GBA1* gene | *GBA1* exons and flanking introns | K(227)R, K79M, G80R, I119L, R120W, S125N, R131C, S173SfsX50, G202R, P246L, Y304C, T323I, R329C, S364N, N370S, G377S, E388K, D409H, L444P, P452L, R463C, R463H, G113A, A446A, RecΔ5, RecNciI (L444P+A456P+V460V), RecA456P (L444P+A456P), c.1263del+RecTL (c.1263–1317del+D409H+L444P+A456P+V460V), D140H, E326K, A190A, Y313Y | K(227)R: PD: 1/1130 (0.09%), Controls: 0/391 (0%)<br>K79M: PD: 0/1130 (0%), Controls: 1/391 (0.26%)<br>G80R: PD: 1/1130 (0.09%), Controls: 0/391 (0%)<br>I119L: PD: 1/1130 (0.09%), Controls: 0/391 (0%)<br>R120W: PD: 1/1130 (0.09%), Controls: 0/391 (0%)<br>S125N: PD: 1/1130 (0.09%), Controls: 0/391 (0%)<br>R131C: PD: 1/1130 (0.09%), Controls: 0/391 (0%)<br>S173SfsX50: PD: 1/1130 (0.09%), Controls: 0/391 (0%)<br>G202R: PD: 2/1130 (0.18%), Controls: 0/391 (0%)<br>P246L: PD: 1/1130 (0.09%), Controls: 0/391 (0%)<br>Y304C: PD: 1/1130 (0.09%), Controls: 0/391 (0%)<br>T323I: PD: 1/1130 (0.09%), Controls: 0/391 (0%)<br>R329C: PD: 2/1130 (0.18%), Controls: 0/391 (0%)<br>S364N: PD: 1/1130 (0.09%), Controls: 0/391 (0%)<br>N370S: PD: 37/1130 (3.27%), Controls: 2/391 (0.51%)<br>G377S: PD: 1/1130 (0.09%), Controls: 0/391 (0%)<br>E388K: PD: 1/1130 (0.09%), Controls: 1/391 (0.26%)<br>D409H: PD: 1/1130 (0.09%), Controls: 0/391 (0%)<br>L444P: PD: 13/1130 (1.15%), Controls: 0/391 (0%) |

| | | | | | | |
|---|---|---|---|---|---|---|
| | | | | | | P452L: PD: 1/1130 (0.09%), Controls: 0/391 (0%)<br>R463C: PD: 1/1130 (0.09%), Controls: 0/391 (0%)<br>R463H: PD: 1/1130 (0.09%), Controls: 0/391 (0%)<br>G113A/A446A: PD: 1/1130 (0.09%), Controls: 0/391 (0%)<br>RecΔ5: PD: 2/1130 (0.18%), Controls: 0/391 (0%)<br>RecNciI (L444P+A456P+V460V): PD: 2/1130 (0.18%), Controls: 0/391 (0%)<br>RecA456P (L444P+A456P): PD: 1/1130 (0.09%), Controls: 0/391 (0%)<br>c.1263del+RecTL (c.1263–1317del+D409H+L444P+A456P+V460V): PD: 1/1130 (0.09%), Controls: 0/391 (0%)<br>A190A: PD: 1/1130 (0.09%), Controls: 0/391 (0%)<br>Y313Y: PD: 0/1130 (0%), Controls: 1/391 (0.26%)<br>E326K: PD: 49/1130 (4.34%), Controls: 8/391 (2.04%)<br>T369M: PD: 17/1130 (1.50%), Controls: 1/391 (0.26%) |
| Spataro et al., 2017 (16) (28124432) | 249/145 | Spanish | Targeted resequencing and CNF detection by eXome- Hidden Markov Model (XHMM) software | NA | N370S | N370S: PD: 1/249 (0.4%), Controls: 0/145 (0%) |
| Setó-Salvia et al., 2012 (17) (22173904) | 225/186 | Spanish | Cycle sequencing of *GBA1* coding region | All *GBA1* exons | N370S, L444P, L144V, S488T, M123T, G202R, I260T, T369M, W393R, D409H, RecNcil | N370S: PD: 5/225 (2.22%), Controls: 0/186 (0%)<br>L444P: PD: 6/225 (2.67%), Controls: 0/186 (0%)<br>L144V: PD: 1/225 (0.44%), Controls: 0/186 (0%)<br>S488T: PD: 1/225 (0.44%), Controls: 0/186 (0%)<br>M123T: PD: 1/225 (0.44%), Controls: 0/186 (0%)<br>G202R: PD: 1/225 (0.44%), Controls: 0/186 (0%)<br>I260T: PD: 1/225 (0.44%), Controls: 0/186 (0%)<br>T369M: PD: 2/225 (0.89%), Controls: 1/186 (0.5%)<br>W393R: PD: 1/225 (0.44%), Controls: 0/186 (0%)<br>D409H: PD: 2/225 (0.89%), Controls: 0/186 (0%)<br>RecNcil: PD: 1/225 (0.44%), Controls: 0/186 (0%) |
| Jesús et al., 2016 (18) (28030538) | 532/542 | Southern Spanish | High-resolution melting (HRM) analysis and direct DNA resequencing | Full *GBA1* gene | N370S, L444P, W312R, V457D, E326K, T369M, etc. | All variants: PD: (12.2%), Controls: (7.9%)<br>N370S: PD: 5/532 (0.94%), Controls: 0/542 (0%)<br>L444P: PD: 13/532 (2.44%), Controls: 6/542 (1.11%)<br>W312R: PD: 6/532 (1.13%), Controls: 2/542 (0.37%)<br>V457D: PD: 3/532 (0.56%), Controls: 4/542 (0.74%)<br>c.116-8C>T: PD: 4/532 (0.75%), Controls: 7/542 (1.29%)<br>E326K: PD: 16/532 (3.00%), Controls: 13/542 (2.40%)<br>T369M: PD: 5/532 (0.94%), Controls: 2/542 (0.37%) |
| Bras et al., 2009 (19) (18160183) | 230/430 | Portuguese | DNA sequencing of the complete open-reading frame, as well as intron/exon boundaries, of the *GBA1* gene | All coding exons and exon/intron boundaries of the *GBA1* gene | N409S, N435T, D448H, L483P, K13R, R41L, E365K, T408M, E427K | Pathogenic variants: PD: 14/230 (6.1%), Controls: 3/430 (0.7%)<br>N409S: PD: 5/230 (2.2%), Controls: 3/430 (0.7%)<br>N435T: PD: 5/230 (2.2%), Controls: 0/430 (0%)<br>D448H: PD: 1/230 (0.4%), Controls: 0/430 (0%)<br>L483P: PD: 3/230 (1.3%), Controls: 0/430 (0%)<br>K13R: PD: 1/230 (0.4%), Controls: 0/430 (0%)<br>R41L: PD: 0/230 (0%), Controls: 1/430 (0.2%)<br>E365K: PD: 2/230 (0.9%), Controls: 3/430 (0.7%)<br>T408M: PD: 2/230 (0.9%), Controls: 5/430 (1.2%)<br>E427K: PD: 0/230 (0%), Controls: 2/430 (0.5%) |
| Petrucci et al., 2020 (20) (32658388) | 874/0 | Italian | Whole exome sequencing | All *GBA1* exons | D24N, S107L, R120W, R131C, P182L, N188S, G202R, H255Q, D409H, L444P, R463C, W209Gfs*6, R257*, E388K, S196P, G202R, H255Q, D409H, T369M, L444P, A456P, V460V, G46E, G193R, R329C, N370S, E326K, N188K, W184R, I161N, | All: PD: 125/874 (14.3%)<br>N370S: PD: 30/874 (3.43%)<br>L444P: PD: 29/874 (3.32%)<br>E326K: PD: 16/874 (1.83%) |

| | | | | | | |
|---|---|---|---|---|---|---|
| | | | | | K(-27)R, M85V, E326D, T369T, V460L | |
| De Marco et al., 2008 (21) (18074383) | 395/483 | Italian | Genotyping | L444P, N370S | L444P, N370S | All: PD: 11/395 (2.8%), Controls: 1/483 (0.2%)<br>L444P: PD: 8/395 (2.0%), Controls: 1/483 (0.2%)<br>N370S: PD: 3/395 (0.8%), Controls: 0/483 (0%) |
| Asselta et al., 2014 (22) (25249066) | 2350/1111 | Italian | High-resolution melting (HRM) analysis (exon 9) and direct DNA sequencing (exon 10) | *GBA1* exons 9 and 10 | IVS8-24T>G, N370S, E388K, IVS9+32C>T, IVS9-36C>G, IVS9-5T>A, D443N, L444P, IVS10+1G>T, IVS10+8C>A | N370S or D443N or L444P or IVS10+1G>T: PD: 106/2350 (4.5%), Controls: 7/1111 (0.63%)<br>N370S: PD+DLB+MSA+PSP+CBD: 69/2766 (2.5%), Controls: 4/1111 (0.36%)<br>L444P: PD+DLB+MSA+PSP+CBD: 47/2766 (1.7%), Controls: 3/1111 (0.27%) |
| Cilia et al., 2016 (23) (27632223) | 2843/0 | Italian | Variant screening of *GBA1* exons 9 and 10 | *GBA1* exons 9 and 10 | N370S, L444P, G377S, IVS10+1G>T | N370S: PD: 70/2843 (2.46%)<br>L444P: PD: 54/2843 (1.90%)<br>G377S: PD: 1/2843 (0.04%)<br>IVS10+1G>T: PD: 1/2843 (0.04%) |
| Straniero et al., 2020 (24) (33209983) | 3691/7757 (1625 partners and caregivers of patients with PD) | Italian | Variant screening | E326K, T369M, N370S, L444P | E326K, T369M, N370S, L444P | E326K: PD: 61/3691 (1.65%), Controls: 55/7755 (0.71%)<br>T369M: PD: 49/3691 (1.33%), Controls: 61/7755 (0.79%)<br>N370S: PD: 76/3691 (2.06%), Controls: 43/7755 (0.55%)<br>L444P: PD: 62/3691 (1.68%), Controls: 11/7755 (0.14%) |
| Quadri et al., 2015 (25) (25294124) | 100/0 | Sardinian | Whole exome sequencing | All *GBA1* exons | N370S, R131C | N370S: PD: 4/100 (4%)<br>R131C: PD: 2/100 (2%) |
| Kalinderi et al., 2009 (26) (19383421) | 172/132 | Greek | DNA sequencing of all *GBA1* exons | All *GBA1* exons | L444P, D409H, E326K, H255Q, R329H, L268L, S271G, T428K, V460L | L444P: PD: 2/172 (1.2%), Controls: 0/132 (0%)<br>D409H: PD: 1/172 (0.6%), Controls: 0/132 (0%)<br>E326K: PD: 1/172 (0.6%), Controls: 1/132 (0.8%)<br>H255Q: PD: 4/172 (2.3%), Controls: 0/132 (0%)<br>R329H: PD: 1/172 (0.6%), Controls: 0/132 (0%)<br>L268L+S271G: PD: 1/172 (0.6%), Controls: 0/132 (0%)<br>T428K: PD: 1/172 (0.6%), Controls: 1/132 (0.8%)<br>V460L: PD: 0/172 (0%), Controls: 4/132 (3.0%) |
| Moraitou et al., 2011 (27) (21745757) | 205/206 | Greek | Restriction enzyme analysis for eight variants | N370S, D409H, L444P, H255Q, R120W, Y108C, IVS10-1G>A, IVS6-2A>G | N370S, D409H, L444P, H255Q, Y108C, IVS10-1G>A, IVS6-2A>G | N370S: PD: 6/205 (2.93%), Controls: 4/206 (1.94%)<br>D409H: PD: 7/205 (3.41%), Controls: 0/206 (0%)<br>L444P: PD: 6/205 (2.93%), Controls: 1/206 (0.49%)<br>H255Q: PD: 7/205 (3.41%), Controls: 1/206 (0.49%)<br>Y108C: PD: 0/205 (0%), Controls: 1/206 (0.49%)<br>IVS10-1G>A: PD: 1/205 (0.49%), Controls: 0/206 (0%) |
| Emekli et al., 2021 (28) (34781237) | 82/0 | Turkish | Next-generation sequencing | All *GBA1* exons and intron/exon boundaries | R434P, H294Q, D448H, G241R, N227K | R434P: PD: 2/82 (2.4%)<br>H294Q: PD: 1/82 (1.2%)<br>D448H: PD: 1/82 (1.2%)<br>G241R: PD: 1/82 (1.2%)<br>N227K: PD: 1/82 (1.2%) |
| Kumar et al., 2013 (29) (22812582) | 360/348 | Serbian | DNA sequencing of *GBA1* exons 8-11 | *GBA1* exons 8-11 | N370S, D409H, H255Q, L444P, A456P, R463C, RecNciI, T369M, E388K, D380V, N392S, V459V | All: PD: 21/360 (5.8%), Controls: 5/348 (1.4%)<br>N370S: PD: 9/360 (2.5%), Controls: 0/348 (0%)<br>D409H, H255Q: PD: 7/360 (1.9%), Controls: 2/348 (0.6%)<br>L444P: PD: 2/360 (0.6%), Controls: 1/348 (0.3%)<br>A456P: PD: 0/360 (0%), Controls: 1/348 (0.3%)<br>R463C: PD: 1/360 (0.3%), Controls: 0/348 (0%)<br>RecNciI (L444P+A456P+V460V): PD: 1/360 (0.3%), Controls: 0/348 (0%)<br>T369M: PD: 8/360 (2.08%), Controls: 6/348 (1.72%)<br>E388K: PD: 0/360 (0%), Controls: 1/348 (0.3%)<br>D380V: PD: 1/360 (0.3%), Controls: 0/348 (0%)<br>N329S: PD: 1/360 (0.3%), Controls: 0/348 (0%)<br>V459V: PD: 0/360 (0%), Controls: 1/348 (0.3%) |
| Török et al., 2016 (30) (26547032) | 124/122 | Hungarian | Variant screening | L444P, N370S, R120W | L444P | L444P: PD: 3/124 (2.4%), Controls: 0/122 (0%) |
| Benitez et al., 2016 (31) (27094865) | 478/337 | European-American | Deep-sequencing of all *GBA1* exons | All *GBA1* exons | R83C, H294Q, T336S, E365K, T408M, N409S, E427K, D448H, L483P, A495P | R83C: PD: 2/478 (0.41%), Controls: 0/337 (0%)<br>H294Q: PD: 2/478 (0.41%), Controls: 0/337 (0%)<br>T336S: PD: 1/478 (0.21%), Controls: 0/337 (0%) |

| | | | | | | |
|---|---|---|---|---|---|---|
| | | | | | | E365K: PD: 19/478 (3.97%), Controls: 11/337 (3.26%)<br>T408M: PD: 17/478 (3.56%), Controls: 0/337 (0%)<br>N409S: PD: 7/478 (1.46%), Controls: 1/337 (0.30%)<br>E427K: PD: 1/478 (0.21%), Controls: 0/337 (0%)<br>D448H: PD: 1/478 (0.21%), Controls: 1/337 (0.30%)<br>L483P: PD: 7/478 (1.46%), Controls: 2/337 (0.59%)<br>A495P: PD: 17/478 (3.56%), Controls: 10/337 (2.97%) |
| Noreau et al., 2011 (32)<br>(21856586) | 212/189 | French-Canadian | Sequencing of the entire coding region of *GBA1* | All *GBA1* exons | L197F, E326K, S339L, T369M, N370S, W378G, L444P | L197F: PD: 1/212 (0.47%), Controls: 0/189 (0%)<br>E326K: PD: 5/212 (2.36%), Controls: 3/189 (1.59%)<br>S339L: PD: 1/212 (0.47%), Controls: 0/189 (0%)<br>T369M: PD: 9/212 (4.25%), Controls: 5/189 (2.65%)<br>N370S: PD: 0/212 (0%), Controls: 2/189 (1.06%)<br>W378G: PD: 1/212 (0.47%), Controls: 0/189 (0%)<br>L444P: PD: 5/212 (2.36%), Controls: 1/189 (0.53%) |
| Han et al., 2016 (33)<br>(26000814) | 225/110<br>(spouses) | Canadian | DNA sequencing of full *GBA1* gene | All *GBA1* exons and flanking introns | c.-119A/G, S(-35)N, R120W, N370S, L444P, RecNciI, RecTL (del55/D409H/RecNciI), E326K, T369M, S13L | All: PD: 25/225 (11.11%), Controls: 9/110 (8.19%)<br>c.-119A/G: PD: 1/225 (0.44%), Controls: 0/110 (0%)<br>S(-35)N: PD: 1/225 (0.44%), Controls: 0/110 (0%)<br>R120W: PD: 1/225 (0.44%), Controls: 0/110 (0%)<br>N370S: PD: 2/225 (0.89%), Controls: 0/110 (0%)<br>L444P: PD: 4/225 (1.78%), Controls: 0/110 (0%)<br>RecNciI (L444P-A456P-V460V): PD: 1/225 (0.44%), Controls: 0/110 (0%)<br>RecTL (del55/D409H/RecNciI): PD: 2/225 (0.89%), Controls: 0/110 (0%)<br>E326K: PD: 4/225 (1.78%), Controls: 4/110 (3.64%)<br>T369M: PD: 11/225 (4.89%), Controls: 4/110 (3.64%)<br>S13L: PD: 0/225 (0%), Controls: 1/110 (0.91%) |
| Sato et al., 2005 (34)<br>(15517592) | 88/122 | Canadian | Genotyping | N370S, L444P, IVS2+1, K198T, R329C, 84insGG, Rec | N370S, L444P, Rec | N370S: PD: 1/88 (1.14%), Controls: 1/122 (0.82%)<br>L444P: PD: 1/88 (1.14%), Controls: 0/122 (0%)<br>Rec: PD: 3/88 (3.41%), Controls: 0/122 (0.%) |
| González-Del Rincón et al., 2013 (35)<br>(23448517) | 128/252 (128 sex and age matched, 124 (aged >60)) | Mexican Mestizo | Variant screening | N370S, L444P | L444P | L444P: PD: 7/128 (5.47%), Controls: 0/252 (0%) |
| Tipton et al., 2020 (36)<br>(32197197) | 209/58 | Colombian and Hispanic American | Variant screening | K198E | K198E | Colombian:<br>K198E: PD: 3 (2.1%), Controls: 1 (1.7%)<br>Hispanic American:<br>K198E: PD: 0 (0%) |
| Velez-Pardo et al., 2019 (37)<br>(30765263) | 602/319 | Colombian, Peruvian | DNA sequencing of all *GBA1* exons and intron/exon boundaries | All *GBA1* exons and intron/exon boundaries | R86X, R159W, R170C, G234W, K237E, N409S, L483P, L483P + RecG or L483P + Rec6b (L483P, +92G>A), Rec1 (L483P, A495P, V499V), RecD, E, or AZRecTL (L483P, A495P, V499V, +92G>A), D66H, R250K, R316H, M400I, E427K, D482N, I528V, R534H, K13R, E365K, T408M | Colombian:<br>R86X: PD: 0/131 (0%), Controls: 0/164 (0%)<br>R159W: PD: 0/131 (0%), Controls: 0/164 (0%)<br>R170C: PD: 0/131 (0%), Controls: 0/164 (0%)<br>G234W: PD: 0/131 (0%), Controls: 1/164 (0.6%)<br>K237E: PD: 7/131 (5.3%), Controls: 2/164 (1.2%)<br>N409S: PD: 3/131 (2.3%), Controls: 0/164 (0%)<br>L483P: PD: 3/131 (2.3%), Controls: 0/164 (0%)<br>L483P + RecG or L483P + Rec6b (L483P, +92G>A): PD: 0/131 (0%), Controls: 0/164 (0%)<br>Rec1 (L483P, A495P, V499V): PD: 0/131 (0%), Controls: 0/164 (0%)<br>RecD, E, or AZRecTL (L483P, A495P, V499V, +92G>A): PD: 0/131 (0%), Controls: 0/164 (0%)<br>D66H: PD: 0/131 (0%), Controls: 0/164 (0%)<br>R250K: PD: 0/131 (0%), Controls: 1/164 (0.6%)<br>R316H: PD: 0/131 (0%), Controls: 0/164 (0%)<br>M400I: PD: 0/131 (0%), Controls: 0/164 (0%)<br>E427K: PD: 0/131 (0%), Controls: 1/164 (0.6%)<br>D482N: PD: 0/131 (0%), Controls: 0/164 (0%)<br>I528V: PD: 0/131 (0%), Controls: 0/164 (0%)<br>R534H: PD: 0/131 (0%), Controls: 1/164 (0.6%) |

| | | | | | | |
|---|---|---|---|---|---|---|
| | | | | | | K13R: PD: 1/131 (0.8%), Controls: 1/164 (0.6%)<br>E365K: PD: 2/131 (1.5%), Controls: 1/164 (0.6%)<br>T408M: PD: 0/131 (0%), Controls: 0/164 (0%)<br><br>Peruvian:<br>R86X: PD: 1/471 (0.2%), Controls: 0/155 (0%)<br>R159W: PD: 2/471 (0.4%), Controls: 0/155 (0%)<br>R170C: PD: 3/471 (0.6%), Controls: 0/155 (0%)<br>G234W: PD: 0/471 (0%), Controls: 0/155 (0%)<br>K237E: PD: 0/471 (0%), Controls: 0/155 (0%)<br>N409S: PD: 1/471 (0.2%), Controls: 1/155 (0.6%)<br>L483P: PD: 7/471 (1.5%), Controls: 0/155 (0%)<br>L483P + RecG or L483P + Rec6b (L483P, +92G>A): PD: 1/471 (0.2%), Controls: 0/155 (0%)<br>Rec1 (L483P, A495P, V499V): PD: 4/471 (0.8%), Controls: 1/155 (0.6%)<br>RecD, E, or AZRecTL (L483P, A495P, V499V, +92G>A): PD: 1/471 (0.2%), Controls: 0/155 (0%)<br>D66H: PD: 1/471 (0.2%), Controls: 0/155 (0%)<br>R250K: PD: 0/471 (0%), Controls: 0/155 (0%)<br>R316H: PD: 1/471 (0.2%), Controls: 0/155 (0%)<br>M400I: PD: 0/471 (0%), Controls: 1/155 (0.6%)<br>E427K: PD: 0/471 (0%), Controls: 0/155 (0%)<br>D482N: PD: 1/471 (0.2%), Controls: 0/155 (0%)<br>I528V: PD: 1/471 (0.2%), Controls: 0/155 (0%)<br>R534H: PD: 0/471 (0%), Controls: 0/155 (0%)<br>K13R: PD: 3/471 (0.6%), Controls: 0/155 (0%)<br>E365K: PD: 5/471 (1.1%), Controls: 0/155 (0%)<br>T408M: PD: 3/471 (0.6%), Controls: 0/155 (0%) |
| Eblan et al., 2006 (38) (16261622) | 33/31 | Venezuelan | DNA sequencing of all *GBA1* exons and most flanking introns | All *GBA1* exons and most flanking introns | N370S, L444P, RecNciI, D443N | N370S: PD: 1/33 (3.0%), Controls: 0/31 (0%)<br>L444P: PD: 1/33 (3.0%), Controls: 0/31 (0%)<br>RecNciI: PD: 2/33 (6.1%), Controls: 0/31 (0%)<br>D443N: PD: 0/33 (0%), Controls: 1/31 (3.32%) |
| Dos Santos et al., 2010 (39) (20816920) | 110/155 | Brazilian | Variant screening | N370S, L444P, 84GG, IVS2+1G>A, G377S | N370S, L444P, D409H+L444P+A456P+V460V, IVS2+1G>A | N370S: PD: 2/110 (1.8%), Controls: 0/155 (0%)<br>L444P: PD: 2/110 (1.8%), Controls: 0/155 (0%)<br>D409H+L444P+A456P+V460V: PD: 1/110 (0.9%), Controls: 0/155 (0%)<br>IVS2+1G>A: PD: 1/110 (0.9%), Controls: 0/155 (0%) |
| Spitz et al., 2008 (40) (17703984) | 65/267 | Brazilian | Variant screening | L444P, N370S | L444P | L444P: PD: 2/65 (3.1%), Controls: 0/267 (0%) |
| Guimarães Bde et al., 2012 (41) (22192918) | 237/186 | Brazilian | Direct sequencing | N370S, L444P | N370S, L444P | L444P: PD: 3/237 (1.27%)<br>N370S: PD: 6/237 (2.53%) |
| Socal et al., 2009 (42) (18358758) | 62/0 | Brazilian (with mixed ethnic backgrounds) | Variant screening | L444P, N370S, IVS2+1, 84GG | L444P, N370S | L444P: PD: 1/62 (1.61%)<br>N370S: PD: 1/62 (1.61%) |
| Barkhuizen et al., 2017 (43) (28361101) | 105/40 | Caucasian/South African (82.7% Afrikaner) | Sanger sequencing of *GBA1* exons 8-11 in all participants; direct sequencing of all *GBA1* exons in 20 PD cases | All *GBA1* exons; *GBA1* exons 8-11 | G35A, E326K, I368T, T369M, N370S, P387L, K441N | G35A: PD: 1/20 (5.0 %)<br>E326K: PD: 5/105 (4.8%), Controls: 1/40 (2.5%)<br>I368T: PD: 1/105 (1.0%), Controls: 0/40 (0%)<br>T369M: PD: 2/105 (1.9%), Controls: 1/40 (2.5%)<br>N370S: PD: 1/105 (1.0%), Controls: 0/40 (0%)<br>P387L: PD: 2/105 (1.9%), Controls: 0/40 (0%)<br>K441N: PD: 1/105 (1.0%), Controls: 0/40 (0%) |
| Mahungu et al., 2020 (44) (32035846) | 30/0 | Black South African | Sanger sequencing of all *GBA1* exons | All *GBA1* exons | K13R, T75del, R159W, R170L, F255L, Q536, G517R, Q471Q | K13R: PD: 6/30 (20%)<br>T75del: PD 2/30 (6.6%)<br>R159W: PD: 1/30 (3.3%)<br>R170L: PD: 1/30 (3.3%)<br>F255L: PD: 6/30 (6.6%)<br>Q536: PD 1/30 (together with F255L) (3.3%) |

| | | | | | | |
|---|---|---|---|---|---|---|
| | | | | | | G517R: PD 1/30 (3.3%)<br>Q471Q: PD: 1/30 (3.3%) |
| Lesage et al., 2011 (45) (21242499) | 194/177 | North African (PD: Algeria: n=147, Morocco: n=23, Tunisia: n=14, Libya: n=1, unknown: n=9; Controls: Algeria: n=95, Morocco: n=46) | DNA sequencing of *GBA1* coding regions | All *GBA1* exons | K(-27)R, R131C, N370S, L444P, E326K, RecNciI (A456P/V460V/L444P), D443N, T369M | K(-27)R: PD: 2/194 (1.03%), Controls: 0/177 (0%)<br>R131C: PD: 2/194 (1.03%), Controls: 0/177 (0%)<br>N370S: PD: 2/194 (1.03%), Controls: 0/177 (0%)<br>L444P/E326K: PD: 1/194 (0.52%), Controls: 0/177 (0%)<br>RecNciI (A456P/V460V/L444P): PD: 2/194 (1.03%), Controls: 0/177 (0%)<br>D443N: PD: 0/194 (0%), Controls: 1/177 (0.56%)<br>E326K: PD: 2/194 (1.03%), Controls: 1/177 (0.56%)<br>T369M: PD: 2/194 (1.03%), Controls: 0/177 (0%) |
| Nishioka et al., 2010 (46) (19945510) | 395/372 | North African Arab-Berber | DNA sequencing of all *GBA1* exons | All *GBA1* exons | K13R, K225R, N370S | K13R: PD: 2/33 (3.0%)<br>K225R: PD: 1/33 (6.1%)<br>K13R: PD: 5/155 (familial, including the 33) (3.2%), 9/240 (sporadic) (3.8%), Controls: 16/372 (4.3%)<br>K225R: PD: 2/155 (familial, including the 33) (1.3%), 0/240 (sporadic) (0%), Controls: 0/372 (0%)<br>N370S: PD: 0/155 (familial, including the 33) (0%), 1/240 (sporadic) (0.4%), Controls: 3/372 (0.8%) |
| Emelyanov et al., 2018 (47) (30146349) | 762/400 | Russian | Variant screening | L444P, N370S, E326K, T369M | L444P, N370S, E326K, T369M | L444P: PD: 9/762 (1.1%), Controls: 1/400 (0.1%)<br>N370S: PD: 4/762 (0.5%), Controls: 0/400 (0%)<br>E326K: PD: 14/762 (2.4%), Controls: 5/400 (1.3%)<br>T369M: PD: 15/762 (2.5%), Controls: 4/400 (1.1%) |
| Emelyanov et al., 2012 (48) (21915911) | 330/240 | Russian | Variant screening | L444P, N370S | L444P, N370S | L444P: PD: 6/330 (1.8%), Controls: 1/240 (0.5%)<br>N370S: PD: 3/330 (0.9%), Controls: 0/240 (0%) |
| Mao et al., 2010 (49) (20004703) | 616/411 | Han-Chinese | Variant screening | L444P | L444P | L444P: PD: 20/616 (3.2%), Controls: 1/411 (0.2%) |
| Hu et al., 2010 (50) (20528910) | 328/300 | Han Chinese | Genotyping | N370S | N370S | N370S: PD: 6/328 (1.8%), Controls: 2/300 (0.7%) |
| Zhang et al., 2012 (51) (23286447) | 195/443 | Han Chinese | Genotyping | L444P, N370S, R120W | L444P | L444P: PD: 6/195 (3.08%), Controls: 0/443 (0%) |
| Guo et al., 2015 (52) (25623333) | 1019/1030 | Han-Chinese | Genotyping | L444P | L444P | L444P: PD: 26/1019 (2.7%), Controls: 1/1030 (0.1%) |
| Wang et al., 2014 (53) (24095219) | 1638/0 | Han Chinese | Genotyping | L444P | L444P | L444P: PD: 49/1638 (2.99%) |
| Ren et al., 2022 (54) (34951095) | 737/0 | Chinese | Next-generation sequencing | Full *GBA1* gene | N370S, E326K, T369M, R163Q, L444P, R120W, etc. | All variants: PD: 79/737 (10.72%)<br>Mild (e.g. N370S): PD: 8/737 (1.09%)<br>Severe (e.g. L444P): PD: 28/737 (3.80%)<br>Risk (e.g. E326K): PD: 1/737 (0.14%)<br>Complex (e.g. L444P-A456P-V460V): PD: 7/737 (0.95%)<br>Unknown: PD: 35/737 (4.75%)<br><br>R163Q: PD: 12/737 (1.63%)<br>L444P: PD: 10/737 (1.36%)<br>R120W: PD: 6/737 (0.81%) |
| Yu et al., 2015 (55) (25518742) | 184/130 | Chinese | DNA sequencing of all *GBA1* exons | All *GBA1* exons | R163Q, F213I, E326K, S364S, F347L, V375L, L444P, RecNciI (L444P-A456P-V460V), A456P Q497R, c.334_338delCAGAA, L264I, L314V | All: PD: 16/184 (8.7%), Controls: 2/130 (1.54%)<br>R163Q: PD: 1/184 (0.54%), Controls: 0/130 (0%)<br>F213I: PD: 1/184 (0.54%), Controls: 0/130 (0%)<br>E326K: PD: 1/184 (0.54%), Controls: 0/130 (0%)<br>S364S: PD: 1/184 (0.54%), Controls: 0/130 (0%)<br>F347L: PD: 1/184 (0.54%), Controls: 1/130 (0.77%)<br>V375L: PD: 1/184 (0.54%), Controls: 0/130 (0%)<br>L444P: PD: 2/184 (1.09%), Controls: 0/130 (0%)<br>RecNciI (L444P-A456P-V460V): PD: 3/184 (1.63%), Controls: 0/130 (0%)<br>A456P: PD: 0/184 (0%), Controls: 1/130 (0.77%)<br>Q497R: PD: 1/184 (0.54%), Controls: 0/130 (0%)<br>c.334_338delCAGAA: PD: 1/184 (0.54%), Controls: 0/130 (0%) |

| | | | | | | |
|---|---|---|---|---|---|---|
| | | | | | | L264I: PD: 2/184 (1.09%), Controls: 0/130 (0%)<br>L314V: PD: 1/184 (0.54%), Controls: 0/130 (0%) |
| Sun et al., 2010 (56) (20131388) | 402/413 | Chinese | Variant screening | L444P, F213I, R353W, N370S | L444P | L444P: PD: 11/402 (2.74%), Controls: 0/413 (0%) |
| Wang et al., 2012 (57) (23227814) | 208/298 | Chinese | Variant screening | L444P, N370S, R120W | L444P | L444P: PD: 7/208 (3.4%), Controls: 1/298 (0.3%)<br>N370S: PD: 0/208 (0%), Controls: 0/298 (0%)<br>R120W: PD: 0/208 (0%), Controls: 0/298 (0%) |
| Tan et al., 2007 (58) (17620502) | 331/347 | Chinese | Allelic discrimination using the 5' nuclease activity assay, adapted to detect the L444P and N370S variants | L444P, N370S | L444P | L444P: PD: 8/331 (2.4%), Controls: 0/347 (0%) |
| Li et al., 2020 (59) (32171587) | 240/0 | Chinese | Whole-exome sequencing | All *GBA1* exons | IVS2+1, G202R, D409H, L444P, R163Q, Y205C, V499M | IVS2+1: PD: 1/240 (0.4%)<br>G202R: PD: 1/240 (0.4%)<br>D409H: PD: 2/240 (0.8%)<br>L444P: PD: 1/240 (0.4%)<br>R163Q: PD: 1/240 (0.4%)<br>Y205C: PD: 1/240 (0.4%)<br>V499M: PD: 1/240 (0.4%) |
| Huang et al., 2011 (60) (21338444) | 967/780 (spouses, patients with unrelated diseases, healthy volunteers) | Chinese | DNA sequencing of whole *GBA1* coding region (in 30 PD cases); Genotyping of L444P, D409H, R120W, L174P, Q497R in all participants | All *GBA1* exons; L444P, D409H, R120W, L174P, Q497R | L444P, D409H, RecNciI (L444P-A456P-V460V) | All: PD: 36/967 (3.72%), Controls: 2/780 (0.26%)<br>L444P: PD: 27/967 (2.79%), Controls: 1/780 (0.13%)<br>D409H: PD: 2/967 (0.21%), Controls: 0/780 (0%)<br>RecNciI (L444P-A456P-V460V): PD: 7/967 (0.72%), Controls: 1/780 (0.13%) |
| Zhang et al., 2015 (61) (26421210) | 1147/0 | Chinese | Variant screening | L444P | L444P | L444P: PD: 34/1147 (2.96%) |
| Foo et al., 2014 (62) (24565865) | 1085/9445 | Chinese, Korean | Sequencing of all *GBA1* exons in EOPD cases and matching controls; genotyping in LOPD cases and matching controls | All *GBA1* exons | Various, including S350R, V221I, P210fs, c.281+1G>A | Chinese EOPD: All rare and low frequency variants: PD: 4/195 (2.05%), Controls: 3/219 (1.37%)<br>Korean EOPD: All rare and low frequency variants: PD: 18/180 (10.0%), Controls: 6/180 (3.33%)<br><br>Chinese LOPD: All rare and low frequency variants: PD: 0/710 (0%), Controls: 3/9046 (0.03%) |
| Ziegler et al., 2007 (63) (17462935) | 92/92 | Chinese from Taiwan | Direct sequencing of full *GBA1* gene | All *GBA1* exons and flanking introns | L444P, D409H, L174P, Q497R, V460M | L444P: PD: 1/92 (1.1%), Controls: 0/92 (0%)<br>D409H: PD: 1/92 (1.1%), Controls: 0/92 (0%)<br>L174P: PD: 1/92 (1.1%), Controls: 0/92 (0%)<br>Q497R: PD: 1/92 (1.1%), Controls: 0/92 (0%)<br>V460M: PD: 0/92 (0%), Controls: 1/92 (1.1%) |
| Gutti et al., 2008 (64) (18541817) | 184/0 | Chinese from Taiwan | Sequencing of *GBA1* gene | Full *GBA1* gene | L444P, R131S, R163Q, L174P, S271G, D409H, Q497R | L444P: PD: 4/184 (2.17%)<br>R131S: PD: 1/184 (0.54%)<br>R163Q: PD: 1/184 (0.54%)<br>L174P: PD: 1/184 (0.54%)<br>S271G: PD: 1/184 (0.54%)<br>D409H: PD: 1/184 (0.54%)<br>Q497R: PD: 1/184 (0.54%) |
| Wu et al., 2007 (65) (17702778) | 518/339 | Taiwanese | Variant screening | L444P, RecNciI, R120W | L444P, RecNciI, R120W | L444P: PD: 13/518 (2.5%), Controls: 2/339 (0.6%)<br>RecNciI: PD: 2/518 (0.4%), Controls: 2/339 (0.6%)<br>R120W: PD: 1/518 (0.2%), Controls: 0/339 (0%) |

| | | | | | | |
|---|---|---|---|---|---|---|
| Choi et al., 2012 (66) (22387070) | 277/291 | Korean | Direct DNA sequencing of all *GBA1* exons in 277 PD cases and 100 controls, only exon 2 and exons 5–11 in 191 controls | All *GBA1* exons; *GBA1* exons 2, 5-11 | I-20V, R163Q, N188S, P201H, R257Q, L268L, S271G, R277C, F347L, L444P, K466K | I-20V: PD: 1/277 (0.36%), Controls: 4/291 (1.37%)<br>R163Q: PD: 1/277 (0.36%), Controls: 0/291 (0%)<br>N188S: PD: 1/277 (0.36%), Controls: 0/291 (0%)<br>P201H: PD: 1/277 (0.36%), Controls: 0/291 (0%)<br>R257Q: PD: 3/277 (1.08%), Controls: 0/291 (0%)<br>L268L: PD: 1/277 (10.36%), Controls: 0/291 (0%)<br>S271G: PD: 2/277 (0.72%), Controls: 0/291 (0%)<br>R277C: PD: 1/277 (0.36%), Controls: 0/291 (0%)<br>F347L: PD: 1/277 (0.36%), Controls: 0/291 (0%)<br>L444P: PD: 2/277 (0.72%), Controls: 0/291 (0%)<br>K466K: PD: 4/277 (1.44%), Controls: 1/291 (0.34%) |
| Li et al., 2014 (67) (24126159) | 147/100 | Japanese | DNA sequencing of all *GBA1* exons and intron/exon boundaries | All *GBA1* exons and exon/intron boundaries of *GBA1* | I(-20)V, G64V, R120W, D409H, L444P, I489V, W393X, K466K, c.1447-1466delTGins, RecNciI | I(-20)V: PD: 13/144 (9.0%), Controls: 10/100 (10.0%)<br>G64V: PD: 1/144 (0.7%), Controls: 0/100 (0%)<br>R120W: PD: 9/144 (6.3%), Controls: 0/100 (0%)<br>D409H: PD: 4/144 (2.8%), Controls: 0/100 (0%)<br>L444P: PD: 12/144 (8.3%), Controls: 0/100 (0%)<br>I489V: PD: 2/144 (1.4%), Controls: 0/100 (0%)<br>W393X: PD: 1/144 (0.7%), Controls: 0/100 (0%)<br>c.1447-1466delTGins: PD: 1/144 (0.7%), Controls: 0/100 (0%)<br>RecNciI: PD: 1/144 (0.7%), Controls: 1/100 (1.0%) |
| Mitsui et al., 2009 (68) (19433656) | 534/544 | Japanese | DNA sequencing of all *GBA1* exons | All *GBA1* exons | R120W, R131C, N188S, R120W-N188R-V191G-S196P-F213I, G193W, F213I, R329C, L444P, L444P-A456P-V460V (RecNciI), A456P-V460V, R496C, I(-20), L(-15)F, L67Q, V121V, D153N, R163Q, P299T, G307S, T334I, L336L, G344G, F347L, R359L, V460V, K466K, I489V | R120W: PD: 15/534 (2.8%), Controls: 0/544 (0%)<br>R131C: PD: 1/534 (0.2%), Controls: 0/544 (0%)<br>N188S: PD: 4/534 (0.7%), Controls: 0/544 (0%)<br>R120W-N188R-V191G-S196P-F213I: PD: 1/534 (0.2%), Controls: 0/544 (0%)<br>G193W: PD: 1/534 (0.2%), Controls: 0/544 (0%)<br>F213I: PD: 1/534 (0.2%), Controls: 0/544 (0%)<br>R329C: PD: 2/534 (0.4%), Controls: 0/544 (0%)<br>L444P: PD: 8/534 (1.5%), Controls: 0/544 (0%)<br>L444P-A456P-V460V (RecNciI): PD: 14/534 (2.6%), Controls: 2/544 (0.4%)<br>A456P-V460V: PD: 1/534 (0.2%), Controls: 0/544 (0%)<br>R496C: PD: 2/534 (0.4%), Controls: 0/544 (0%)<br>I(-20)V: PD: 77/534 (14.4%), Controls: 66/544 (12.1%)<br>L(-15)F: PD: 1/534 (0.2%), Controls: 0/544 (0%)<br>L67Q: PD: 0/534 (0%), Controls: 1/544 (0.2%)<br>V121V: PD: 0/534 (0%), Controls: 1/544 (0.2%)<br>D153N: PD: 1/534 (0.2%), Controls: 0/544 (0%)<br>R163Q: PD: 4/534 (0.7%), Controls: 7/544 (1.3%)<br>P299T: PD: 1/534 (0.2%), Controls: 0/544 (0%)<br>G307S: PD: 1/534 (0.2%), Controls: 0/544 (0%)<br>T334I: PD: 0/534 (0%), Controls: 1/544 (0.2%)<br>L336L: PD: 1/534 (0.2%), Controls: 0/544 (0%)<br>G344G: PD: 1/534 (0.2%), Controls: 0/544 (0%)<br>F347L: PD: 0/534 (0%), Controls: 1/544 (0.2%)<br>R359L: PD: 1/534 (0.2%), Controls: 0/544 (0%)<br>V460V: PD: 2/534 (0.4%), Controls: 1/544 (0.2%)<br>K466K: PD: 11/534 (2.1%), Controls: 8/544 (1.5%)<br>I489V: PD: 4/534 (0.7%), Controls: 3/544 (0.6%) |
| Pulkes et al., 2014 (69) (24997549) | 480/395 | Thai | Direct DNA sequencing in all EOPD and 100 patients with AAO>50; Variant screening in remaining patients with AAO>50 and controls | All *GBA1* exons and exon/intron boundaries of *GBA1* | L444P, N386K, P428S, IVS2+1G>A, IVS9+3G>C, IVS10-9_10GT>AG, V398fsX404 | L444P: PD: 15/480 (3.1%), EOPD: 8/108 (7.4%), AAO>50: 7/372 (1.9%), Controls: 1/395 (0.3%)<br>N386K: PD: 1/480 (0.2%), EOPD: 1/108 (0.9%), AAO>50: 0/372 (0%), Controls: 0/395 (0%)<br>P428S: PD: 2/480 (0.4%), EOPD: 1/108 (0.9%), AAO>50: 1/372 (0.3%), Controls: 1/395 (0.3%)<br>IVS2+1G>A: PD: 1/480 (0.2%), EOPD: 1/108 (0.9%), AAO>50: 0/372 (0%), Controls: 0/395 (0%)<br>IVS9+3G>C: PD: 1/480 (0.2%), EOPD: 1/108 (0.9%), AAO>50: 0/372 (0%), Controls: 0/395 (0%) |

| | | | | | |
|---|---|---|---|---|---|
| | | | | | IVS10-9_10GT>AG: PD: 3/480 (0.6%), EOPD: 1/108 (0.9%), AAO>50: 2/372 (0.5%), Controls: 0/395 (0%)<br>V398fsX404: PD: 1/480 (0.2%), EOPD: 1/108 (0.9%), AAO>50: 0/372 (0%), Controls: 0/395 (0%) |
| Yadav et al., 2018 (70) (30504558) | 100/0 | Indian | DNA sequencing of all *GBA1* exons and intron/exon boundaries | All *GBA1* exons and exon/intron junctions | IVS1+191G>C, IVS4+47G>A, IVS6−86A>G, IVS9+141A>G, IVS10+3G>A | IVS1+191G>C: PD 1/100 (1%)<br>IVS4+47G>A: PD 64/100 (64%)<br>IVS6−86A>G: PD 65/100 (65%)<br>IVS9+141A>G: PD 65/100 (65%)<br>IVS10+3G>A: PD 1/100 (1%) |
| Biswas et al., 2021 (71) (33711404) | 198/241 | Indian | Variant screening | IVS2+1A>G, R120W, H255Q, R257Q, E326K, N370S, D409H, L444P, RecNciI | L444P | L444P: PD: 2/198 (1.01%), Controls: 0/241 (0%) |
| Halder et al., 2016 (72) (NA) | 114/120 | Indian | Variant screening | L444P, N370S | L444P | L444P: PD: 4/114 (3.51%), Controls: 0/120 (0%) |
| Goldstein et al., 2019 (73) (31662221) | 1200/378 | Ashkenazi Jewish | Genotyping | E326K, T369M, R44C, N370S, R496H, L444P, 84GG, IVS2+1G->A, V394L, Rec370 | E326K, T369M, R44C, N370S, R496H, L444P, 84GG, IVS2+1G->A, V394L, Rec370 | E326K: PD: 17/1200 (1.4%), Controls: 5/378 (1.32%)<br>R44C: PD: 1/1200 (0.08%), Controls: 0/378 (0%)<br>T369M: PD: 11/1200 (0.92%), Controls: 1/378 (0.26%)<br>N370S or R535H: PD: 140/1200 (11.67%), Controls: 14/378 (3.7%)<br>84GG or IVS2+1 or V349L or L444P or Rec370: PD: 46/1200 (3.83%), Controls: 2/378 (0.53%) |
| Gan-Or et al., 2008 (74) (18434642) | 420/4138 | Ashkenazi Jewish | Variant screening | N370S, R496H, 84GG IVS2+1, V394L, D409H, L444P, RecTL | N370S, R496H, 84GG, IVS2+1, V394L, D409H, L444P, RecTL | All: PD: 75/420 (17.9%), Elderly controls: (4.2%), Young controls: (6.35%)<br>N370S: PD: 46/420 (10.95%), Elderly controls: 11/333 (3.3%), Young controls: 224/3805 (5.89%)<br>R496H: PD: 7/420 (1.67%), Elderly controls: 1/333 (0.3%)<br>84GG: PD: 8/420 (1.90%), Elderly controls: 1/333 (0.3%), Young controls: 6/3805 (0.16%)<br>IVS2+1: PD: 4/420 (0.95%), Young controls: 4/3805 (0.11%)<br>V394L: PD: 3/420 (0.71%), Young controls: 4/3805 (0.11%)<br>L444P: PD: 2/420 (0.48%), Young controls: 2/3805 (0.05%) |
| Gan-Or et al., 2015 (75) (25653295) | 1000/3805 | Ashkenazi Jewish | Variant screening | 84GG, IVS2+1, N370S, L444P, V394L, R496H, 370Rec | 84GG, IVS2+1, N370S, L444P, V394L, R496H, 370Rec | All: PD: 192/1000 (19.2%), Controls: 242/3805 (6.4%)<br>N370S: PD: 131/1000 (13.1%), Controls: 225/3805 (5.9%)<br>R496H: PD: 19/1000 (1.9%), Controls: NT<br>84GG: PD: 21/1000 (2.1%), Controls: 6/3805 (0.16%)<br>IVS2+1G>A: PD: 5/1000 (0.5%), Controls: 1/3805 (0.03%)<br>V394L: PD: 11/1000 (1.1%), Controls: 4/3805 (0.11%)<br>L444P: PD: 3/1000 (0.3%), Controls: 4/3805 (0.11%)<br>370Rec: PD: 10/1000 (1.0%), Controls: 2/3805 (0.05%) |
| Dagan et al., 2015 (76) (26169695) | 287/400 | Ashkenazi Jewish | Variant screening | N370S, L444P, c.84GG, c.115+1G>A (IVS2+1G>A), V394L, R496H | N370S, c.84GG, V394L, R496H | N370S: PD: 54/287 (18.8%), Controls: 14/400 (3.5%)<br>c.84GG: PD: 9/287 (3.1%), Controls: 2/400 (0.5%)<br>V394L: PD: 1/287 (0.3%)<br>R496H: PD: 4/287 (1.4%), Controls: 3/400 (0.7%) |
| Liu et al., 2011 (77) (21812969) | 268/178 | Ashkenazi Jewish | Genotyping | NA | N370S | N370S: PD: 28/268 (10.4%) |
| Ruskey et al., 2019 (78) (29842932) | 735/622 | Ashkenazi Jewish | Targeted next-generation sequencing; Sanger sequencing of exons 10 and 11 | Full *GBA1* gene | 84GG, R44C, N188S, E326K, T369M, N370S, A384D, V394L, T410M, L444P, L461P, R496H | 84GG: PD: 13/735 (1.77%), Controls: 1/622 (0.15%)<br>R44C: PD: 2/735 (0.27%), Controls: 7/622 (1.06%)<br>N188S: PD: 1/735 (0.14%), Controls: 0/622 (0%)<br>E326K: PD: 13/735 (1.77%), Controls: 2/622 (0.3%)<br>T369M: PD: 2/735 (0.27%), Controls: 0/622 (0%)<br>N370S: PD: 92/735 (12.52%), Controls: 37/622 (5.58%)<br>A384D: PD: 1/735 (0.14%), Controls: 0/622 (0%)<br>V394L: PD: 1/735 (0.14%), Controls: 0/622 (0%)<br>T410M: PD: 0/735 (0%), Controls: 1/622 (0.15%)<br>L444P: PD: 3/735 (0.41%), Controls: 0/622 (0%) |

| | | | | | | |
|---|---|---|---|---|---|---|
| | | | | | | L461P: PD: 1/735 (0.14%), Controls: 0/622 (0%)<br>R496H: PD: 9/735 (1.22%), Controls: 2/622 (0.3%) |
| Clark et al., 2005 (79) (15517591) | 160/92 | Ashkenazi Jewish | Direct sequencing | N370S | N370S | N370S: PD: 17/160 (10.6%), Controls: 4/92 (4.3%) |
| Aharon-Peretz et al., 2004 (80) (15525722) | 99/1543 | Ashkenazi Jewish | Variant screening | N370S, L444P, 84GG, IVS+1, V394L, R496H | N370S, 84GG, R496H | N370S: PD: 26/99 (26.3%), Controls: 92/1543 (5.96%)<br>84GG: PD: 4/99 (4.0%), Controls: 3/1543 (0.19%)<br>R496H: PD: 1/99 (1.0%), Controls: 0/1543 (0%) |
| Aharon-Peretz et al., 2005 (81) (16148263) | 148/0 | Ashkenazi Jewish | Digestion with appropriate enzymes to detect N370S, L444P, 84GG, IVS+1, V394L, R496H | N370S, L444P, 84GG, IVS+1, V394L, R496H | N370S,4GG, R496H | N370S: PD: 34/148 (22.97%)<br>84GG: PD: 4/148 (2.70%)<br>R496H: 2/148 (1.35%) |
| Gan-Or et al., 2010 (82) (19458969) | 600/0 | Ashkenazi Jewish | Variant screening | 84GG, IVS2+1, N370S, V394L, D409H, L444P, R496H and RecTL | 84GG, IVS2+1, N370S, V394L, D409H, L444P, R496H and RecTL | All: PD: 117/600 (19.5%) |
| Alcalay et al., 2015 (83) (26117366) | 517/252 (mostly spouses) | Mixed (PD: 231 with Ashkenazi Jewish Grandparent, Controls: 97 with Ashkenazi Jewish Grandparent) | DNA sequencing of the full *GBA1* gene | Full *GBA1* gene | N370S, L444P, 84GG, R496H, IVS2+1, K-27R, E326K, T369M, L461P, V294M, A456P, G241R, Rearrangement exon 8, Q-8H, R44C, N392S, S110A, T410M, F-36V, P387P, E349K | N370S: PD: 36/517 (7.0%), Controls: 4/252 (1.6%)<br>L444P: PD: 7/517 (1.4%), Controls: 1/252 (0.4%)<br>84GG: PD: 4/517 (0.8%), Controls: 0/252 (0%)<br>R496H: PD: 4/517 (0.8%), Controls: 0/252 (0%)<br>IVS2+1: PD: 2/517 (0.4%), Controls: 0/252 (0%)<br>K-27R: PD: 2/517 (0.4%), Controls: 0/252 (0%)<br>E326K: PD: 13/517 (2.5%), Controls: 3/252 (1.2%)<br>T369M: PD: 5/517 (1.0%), Controls: 4/252 (1.6%)<br>L461P: PD: 1/517 (0.2%), Controls: 0/252 (0%)<br>V294M: PD: 1/517 (0.2%), Controls: 0/252 (0%)<br>A456P: PD: 1/517 (0.2%), Controls: 0/252 (0%)<br>G241R: PD: 1/517 (0.2%), Controls: 0/252 (0%)<br>Rearrangement exon 8: PD: 1/517 (0.2%), Controls: 0/252 (0%)<br>Q-8H: PD: 1/517 (0.2%), Controls: 0/252 (0%)<br>R44C: PD: 1/517 (0.2%), Controls: 0/252 (0%)<br>N392S: PD: 1/517 (0.2%), Controls: 0/252 (0%)<br>S110A: PD: 0/517 (0%), Controls: 1/252 (0.4%)<br>T410M: PD: 0/517 (0%), Controls: 1/252 (0.4%)<br>F-36V: PD: 0/517 (0%), Controls: 1/252 (0.4%)<br>P387P: PD: 0/517 (0%), Controls: 1/252 (0.4%)<br>E349K: PD: 0/517 (0%), Controls: 1/252 (0.4%) |
| Clark et al., 2007 (84) (17875915) | 278/179 | Mixed (178 PD Jewish, 85 controls Jewish) | DNA sequencing of all *GBA1* exons | All *GBA1* exons | 84insGG, E326K, T369M, N370S, D409H, R496H, L444P, RecNciI (L444P + A456P + V460V), P175P | All: PD: 38/278 (13.7%), Controls: 8/179 (4.5%)<br>84insGG: PD: 5/278 (1.8%), Controls: 0/179 (0%)<br>E326K: PD: 1/278 (0.4%), Controls: 1/179 (0.6%)<br>T369M: PD: 3/278 (1.1%), Controls: 3/179 (1.7%)<br>N370S: PD: 23/278 (8.3%), Controls: 4/179 (2.2%)<br>D409H: PD: 1/278 (0.4%), Controls: 0/179 (0%)<br>R496H: PD: 1/278 (0.4%), Controls: 0/179 (0%)<br>L444P: PD: 2/278 (0.7%), Controls: 0/179 (0%)<br>RecNciI (L444P + A456P + V460V): PD: 1/278 (0.4%), Controls: 0/179 (0%)<br>P175P: PD: 1/278 (0.4%), Controls: 0/179 (0%) |
| Alcalay et al., 2010 (85) (20837857) | 953/0 | Mixed (77 Hispanics, 139 of Jewish ancestry) | DNA sequencing of full *GBA1* gene in 90 cases (previously reported); Genotyping for L444P and N370S in 515 | Full *GBA1* gene; L444P, N370S | L444P, N370S | L444P: PD: 18/953 (1.9%)<br>N370S: PD: 40/953 (4.2%) |

| | | | | | | |
|---|---|---|---|---|---|---|
| | | | cases; direct sequencing of L444P and N370S in 348 cases | | | |
| Goker-Alpan et al., 2006 (86) (16790605) | 28/44 | Mixed | DNA sequencing of full *GBA1* gene | All *GBA1* exons and flanking introns | N370S | N370S: PD: 1/28 (3.57%), Controls: 0/44 (0%) |
| Sidransky et al., 2009 (87) (19846850) | 5691/4898 | Mixed | Variant screening for L444P, N370S or sequencing for all *GBA1* exons | L444P, N370S; all *GBA1* exons | L444P, N370S, E326K, T369M | Ashkenazi Jews:<br>L444P/N370S: PD (15.3%), Controls (3.4%)<br>Non-Ashkenazi Jews:<br>L444P/N370S: PD (3.2%), Controls (0.6%)<br><br>Center Brazil (N370S, L444P, G377S):<br>PD: 4/65 (6.2%), Controls: 0/264 (0%)<br>Center NYC, USA (Full sequencing):<br>PD: 34/275 (177 AJ) (12.4%), Controls: 3/140. (65 AJ) (2.14%)<br>Center France (N370S, L444P, D409H):<br>PD: 12/297 (4.0%), Controls: 1/251 (0.39%)<br>Center Haifa, IL (D409H, 84GG, V394L, IVS2+1, R496H):<br>PD: 40/162 (162 AJ) (24.7%), Controls: NP<br>Center Italy (L444P, N370S):<br>PD: 11/395 (2.8%), Controls: 1/483 (0.21%)<br>Center Norway (L444P, N370S):<br>PD: 7/311 (2.3%), Controls: 8/473 (1.69%)<br>Center NHGRI, USA (Full sequencing):<br>PD: 29/539 (5.4%), Controls: 6/209 (1 AJ) (2.87%)<br>Center Portugal (Full sequencing):<br>PD: 15/231 (6.5%), Controls: 6/482 (1.24%)<br>Center Rostock, DE (Full Sequencing):<br>PD: 18/298 (6.0%), Controls: 5/212 (2.4%)<br>Center Singapore (L444P, N370S):<br>PD: 8/329 (2.4%), Controls: 0/201 (0%)<br>Center Taiwan (L444P, recNciI, R120W, some full sequencing):<br>PD: 22/559 (3.9%), Controls: 4/377 (1.06%)<br>Center Tel Aviv, IL (84GG, IVS2+1, N370S, V394L, D409H, L444P, R496H, RecTL):<br>PD: 81/420 (419 AJ) (19.3%), Controls: 13/321 (321 AJ) (4.05%)<br>Center Japan (full sequencing):<br>PD: 50/534 (9.4%), Controls: 2/546 (0.37%)<br>Center Tübingen, DE (L444P, N370S):<br>PD: 12/377 (3.2%), Controls: 0/325 (0%)<br>Center Toronto, CA (N370S, K178T, L444P, 84GG, R329C, IVS2+1, recNciiI):<br>PD: 5/88 (2 AJ) (5.7%), Controls: 1/96 (1.0%) |
| Mata et al., 2016 (88) (26296077) | 1369/0 | Mixed | DNA sequencing of all *GBA1* exons and intron-exons boundaries | All *GBA1* exons and intron-exons boundaries | IVS2+1G>A, 84dupG, S125N, T134P, D140H, R163X, N188S, S196P, G202R, F216Y, 914delC, S271G, R359X, N370S, Rec3 (c1263-1317 del, D409H, L444P, A456P, V460V), D409H, L444P, Rec1 (L444P, A456P, V460V), Rec L444P + V460V, V460M, R463C, R496H, R(−32)T, P(−28)S, R44C, G193E, R262H, F316I, G344S, D443N, V460L, S488T, K(−27)R, E326K, T369M | IVS2+1G>A: PD: 2/1369 (0.15%)<br>84dupG: PD: 3/1369 (0.22%)<br>S125N: PD: 1/1369 (0.07%)<br>T134P: PD: 1/1369 (0.07%)<br>D140H: PD: 2/1369 (0.22%)<br>R163X: PD: 1/1369 (0.07%)<br>N188S: PD: 1/1369 (0.07%)<br>S196P: PD: 1/1369 (0.07%)<br>G202R: PD: 1/1369 (0.07%)<br>F216Y: PD: 1/1369 (0.07%)<br>914delC: PD: 1/1369 (0.07%)<br>S271G: PD: 1/1369 (0.07%)<br>R359X: PD: 1/1369 (0.07%) |

| | | | | | | |
|---|---|---|---|---|---|---|
| | | | | | | N370S: PD: 18/1369 (1.31%)<br>Rec3 (c1263-1317 del, D409H, L444P, A456P, V460V): PD: 1/1369 (0.07%)<br>D409H: PD: 1/1369 (0.07%)<br>L444P: PD: 16/1369 (1.17%)<br>Rec1 (L444P, A456P, V460V): PD: 2/1369 (0.22%)<br>Rec L444P + V460V: PD: 1/1369 (0.07%)<br>V460M: PD: 1/1369 (0.07%)<br>R463C: PD: 3/1369 (0.22%)<br>R496H: PD: 2/1369 (0.22%)<br>R(−32)T: PD: 1/1369 (0.07%)<br>P(−28)S: PD: 1/1369 (0.07%)<br>R44C: PD: 1/1369 (0.07%)<br>G193E: PD: 1/1369 (0.07%)<br>R262H: PD: 1/1369 (0.07%)<br>F316I: PD: 1/1369 (0.07%)<br>G344S: PD: 1/1369 (0.07%)<br>D443N: PD: 1/1369 (0.07%)<br>V460L: PD: 1/1369 (0.07%)<br>S488T: PD: 1/1369 (0.07%)<br>K(−27)R: PD: 6/1369 (0.44%)<br>E326K: PD: 69/1369 (5.04%)<br>T369M: PD: 30/1369 (2.19%) |
| Nichols et al., 2009 (89) (18987351) | 1325/359 | International | DNA sequencing of all *GBA1* exons and corresponding intron/exon boundaries in 96 samples; Variant screening in 1325 cases and 359 controls | All *GBA1* exons and intron/exon boundaries | IVS6 589-2A>G, R262H, K303K, E326K, T369M, N370S, L444P, IVS10 1389-3C>G, RecNciI (L444P+A456P+V60V) | IVS6 589-2A>G/R262H/K303K/E326K/T369M/N370S/L444P/IVS10 1389-3C>G/RecNciI (L444P+A456P+V60V): PD: 21/96 (21.9%)<br>All 9 *GBA1* variants: PD: 161/1325 (12.2%)<br>All 5 previous *GBA1* variants (E326K, T369M, N370S, L444P, RecNcil) : PD: ?/450 (12.6%), Controls: ?/359 (5.3%)<br>E326K: PD: ?/450 (6.2%), Controls: ?/359 (3.1%)<br>T369M: PD: ?/450 (2.3%), Controls: ?/359 (1.1%)<br>N370S: PD: ?/450 (1.4%), Controls: ?/359 (0.8%)<br>L444P: PD: ?/450 (1.9%), Controls: ?/359 (0.0%)<br>A456P/V460V/L444P: PD: ?/450 (0.8%), Controls: ?/359 (0.3%)<br>IVS6 589-2A>G: Controls: 0/359 (0%)<br>R262H: Controls: 0/359 (0%)<br>IVS10 1389- 3C>G: Controls: 0/359 (0%) |
| Liu et al., 2016 (90) (27717005) | 2304/0 | International | Depending on the study: Whole exome or targeted sequencing or genotyping of N370S, E326K, T369M | All *GBA1* exons; N370S, E326K, T369M | K(-27)R, 84GG, R120W, D140H, G195E, H255Q, R257Q, P266L, R359X, G377S, D409H, L444P, L444R, A456P, N462K, R463C, R463P, N370S, E326K, T369M, E388K | 84GG: PD: 1/1921 (0.05%)<br>R120W: PD: 1/1921 (0.05%)<br>D140H: PD: 8/1921 (0.42%)<br>G195E: PD: 1/1921 (0.05%)<br>H255Q: PD: 1/1921 (0.05%)<br>R257Q: PD: 2/1921 (0.10%)<br>P266L: PD: 1/1921 (0.05%)<br>R359X: PD: 1/1921 (0.05%)<br>G377S: PD: 1/1921 (0.05%)<br>D409H: PD: 1/1921 (0.05%)<br>L444P: PD: 13/1921 (0.68%)<br>L444R: PD: 1/1921 (0.05%)<br>A456P: PD: 1/1921 (0.05%)<br>N462K: PD: 1/1921 (0.05%)<br>R463C: PD: 2/1921 (0.10%)<br>N370S: PD: 28/1921 (1.5%)<br>E326K: PD: 92/1921 (4.79%)<br>T369M: PD: 48/1921 (2.50%)<br>E388K: PD: 2/1921 (0.10%) |
| Stoker et al., 2020 (91) (32303560) | 250/0 | NA | DNA sequencing of all *GBA1* exons in 250 patients; | All *GBA1* exons | N370S, L444P, R463C, G10S, N426K, R48W, R257Q, c.762 18T>A, E326K, T369M, E388K, L119L, c.589 86A>G) | All: PD: 36/250 (14.4%)<br>N370S: PD: 3/250 (1.2%)<br>L444P: PD: 3/250 (1.2%)<br>R463C: PD: 1/250 (0.4%) |

| | | | | | | |
|---|---|---|---|---|---|---|
| | | | genotyping in 127 patients | | | G10S: PD: 1/250 (0.4%)<br>N426K: PD: 1/250 (0.4%)<br>R48W: PD: 1/250 (0.4%)<br>R257Q: PD: 1/250 (0.4%)<br>c.762 18T>A: PD: 8/250 (3.2%)<br>E326K PD: 7/250 (2.8%)<br>T369M: PD: 7/250 (2.8%)<br>E388K: PD: 1/250 (0.4%)<br>L119L: PD: 1/250 (0.4%)<br>c.589 86A>G: PD: 1/250 (0.4%)<br><br>N370S: PD: 4/127 (3.1%)<br>R463C: PD: 1/127 (0.8%)<br>G10S PD: 1/127 (0.8%)<br>T369M PD: 3/127 (2.4%)<br>E326K PD: 2/127 (1.6%)<br>E388K PD: 1/127 (0.8%) |
| Gorostidi et al., 2016 (92) (27294386) | 92/0 | NA | Targeted DNA sequencing | NA | K13R, Y244C, T408M | K13R: PD: 1/92 (1.1%)<br>Y244C: PD: 1/92 (1.1%)<br>T408M: PD: 3/92 (3.3%) |
| Mata et al., 2008 (93) (18332251) | 721/554 (310 spouses, 244 volunteers) | NA | Genotyping | N370S, L444P | N370S, L444P | N370S: PD: 11/721 (1.5%), Controls: 2/554 (0.4%)<br>L444P: PD: 10/721 (1.4%), Controls: 0/554 (0%) |
| Lwin et al., 2004 (94) (14728994) | 57/44 | NA | DNA sequencing of full *GBA1* gene | All *GBA1* exons and flanking introns | N370S, L444P, K198T, R329C, T369M, E326K | N370S: PD: 5/57 (8.77%), Controls: 0/44 (0%)<br>L444P: PD: 1/57 (1.75%), Controls: 0/44 (0%)<br>K198T: PD: 1/57 (1.75%), Controls: 0/44 (0%)<br>R329C: PD: 1/57 (1.75%), Controls: 0/44 (0%)<br>T369M PD: 3/57 (5.26%), Controls: 0/44 (0%)<br>E326K PD: 1/57 (1.75%), Controls: 0/44 (0%) |
| Malek et al., 2018 (95) (29378790) | 1893/0 | NA | DNA sequencing of all *GBA1* exons | All *GBA1* exons | L444P, N370S, R463C, G202R, R359S, E326K, T369M, D409H, F213I, G189V, G377S, K157Q, L383Xfs, L66P, M123T, N382Xfs, R163s, R257Q, S173s, E481Xfs, G10S, G325W, R170H, T323I, L175I, L324V, P55S, R262H, R329H, R395C, T267I, L268L, Asp315His,<br>Exon 3 hemizygous deletion, A456P, V460V, D140H, I308T, Ex4 hemizygous deletion | L444P: PD: 30/1893 (1.6%)<br>N370S: PD: 11/1893 (0.6%)<br>R463C: PD: 5/1893 (0.3%)<br>G202R: PD: 2/1893 (0.1%)<br>R359S: PD: 2/1893 (0.1%)<br>E326K: PD: 86/1893 (4.5%)<br>T369M: PD: 35/1893 (1.8%)<br>D409H: PD: 1/1893 (0.05%)<br>F213I: PD: 1/1893 (0.05%)<br>G189V: PD: 1/1893 (0.05%)<br>G377S: PD: 1/1893 (0.05%)<br>K157Q: PD: 1/1893 (0.05%)<br>L383Xfs: PD: 1/1893 (0.05%)<br>L66P: PD: 1/1893 (0.05%)<br>M123T: PD: 1/1893 (0.05%)<br>N382Xfs: PD: 1/1893 (0.05%)<br>R163s: PD: 1/1893 (0.05%)<br>R257Q: PD: 1/1893 (0.05%)<br>S173s: PD: 1/1893 (0.05%)<br>E481Xfs: PD: 1/1893 (0.05%)<br>G10S: PD: 1/1893 (0.05%)<br>G325W: PD: 1/1893 (0.05%)<br>R170H: PD: 1/1893 (0.05%)<br>T323I: PD: 1/1893 (0.05%)<br>L175I: PD: 1/1893 (0.05%)<br>L324V: PD: 1/1893 (0.05%)<br>P55S: PD: 1/1893 (0.05%)<br>R262H: PD: 1/1893 (0.05%)<br>R329H: PD: 1/1893 (0.05%) |

| | | | | | | |
|---|---|---|---|---|---|---|
| | | | | | | R395C: PD: 1/1893 (0.05%)<br>T267I: PD: 1/1893 (0.05%)<br>L268L: PD: 1/1893 (0.05%)<br>Asp315His: PD: 1/1893 (0.05%)<br>Exon 3 hemizygous deletion: PD: 1/1893 (0.05%)<br>A456P: PD: 6/1893 (0.3%)<br>V460V: PD: 6/1893 (0.3%)<br>D140H: PD: 2/1893 (0.1%)<br>I308T: PD: 2/1893 (0.1%)<br>Ex4 hemizygous deletion: PD: 2/1893 (0.1%) |
| Eblan et al., 2005 (96)<br>(15716572) | 26/0 | NA | DNA sequencing | NA | D140H, RecNciI | D140H: PD: 1/26 (3.85%)<br>RecNciI: PD: 1/26 (3.85%) |
| Barber et al., 2017 (97)<br>(28472425) | 106/283 | NA | Variant screening | N370S, L444P | N370S | N370S: PD: 1/106 (0.9%), Controls: 1/283 (0.4%) |
| Malec-Litwinowicz et al., 2014<br>(98)<br>(25168325) | 138/0 | NA | DNA sequencing of *GBA1* exons 8 and 9 | *GBA1* exons 8 and 9 | N370S, T369M | N370S: PD: 5/138 (3.6%)<br>T369M: PD: 11/138 (7.9%) |
| McNeill et al., 2012 (99)<br>(22577228) | 220/0 | NA | Sanger sequencing of the full *GBA1* gene | Full *GBA1* gene | N370S, L444P, Recombinant alleles, R496H, V460L, IVS2+1 | N370S: PD: 5/220 (2.27%)<br>L444P: PD: 2/220 (0.91%)<br>Recombinant alleles: PD: 2/220 (0.91%)<br>R496H: PD: 1/220 (0.45%)<br>V460L: PD: 1/220 (0.45%)<br>IVS2+1: PD: 1/220 (0.45%) |
| Graham et al., 2020 (100)<br>(31809948) | 229/50 | NA | Nanopore sequencing of full *GBA1* gene | Full *GBA1* gene | E365K, T408M, D179H, N409S, L335=, R78C | E365K: PD: 12/229 (5.24%), Controls: 0/50 (0%)<br>T408M: PD: 7/229 (3.06%), Controls: 2/50 (4.0%)<br>D179H: PD: 1/229 (0.44%), Controls: 0/50 (0%)<br>N409S: PD: 1/229 (0.44%), Controls: 0/50 (0%)<br>L335=: PD: 1/229 (0.44%), Controls: 0/50 (0%)<br>R78C: PD: 1/229 (0.44%), Controls: 0/50 (0%) |

| **Population** | **Variants** | **n mutation carriers/n total (frequency) in PD** | **n mutation carriers/n total (frequency) in controls** | **n studies included** | **References** |
|---|---|---|---|---|---|
| White/Caucasian | p.L483P<br>p.N409S<br>p.T408M<br>p.E365K<br>p.R159W | 329/21492 (1.53%)<br>345/21714 (1.59%)<br>310/15266 (2.03%)<br>668/16408 (4.07%)<br>8/10011 (0.08%) | 101/17770 (0.57%)<br>76/17770 (0.43%)<br>182/14298 (1.27%)<br>239/14849 (1.61%)<br>0/4630 (0%) | 34 | (1), (2), (3), (4), (5), (6), (7), (8), (9), (10), (11), (12), (13), (14), (15), (16), (17), (18), (19), (20), (21), (22), (23), (24), (25), (26), (27), (28), (29), (30), (31), (32), (33), (34) |
| South American | p.L483P<br>p.N409S<br>p.T408M<br>p.E365K<br>p.R159W | 26/1237 (2.10%)<br>14/1237 (1.13%)<br>3/635 (0.47%)<br>7/635 (1.10%)<br>2/635 (0.31%) | 0/1024 (0%)<br>1/1024 (0.10%)<br>0/350 (0%)<br>1/350 (0.29%)<br>0/350 (0%) | 7 | (35), (37), (38), (39), (40), (41), (42) |
| North African | p.L483P<br>p.N409S<br>p.T408M<br>p.E365K<br>p.R159W | 1/227 (0.44%)<br>3/589 (0.51%)<br>2/227 (0.88%)<br>3/227 (1.32%)<br>0/227 (0%) | 0/177 (0%)<br>3/549 (0.55%)<br>0/177 (0%)<br>1/177 (0.56%)<br>0/177 (0%) | 2 | (45), (46) |
| Asian | p.L483P<br>p.N409S<br>p.T408M<br>p.E365K<br>p.R159W | 261/10233 (2.55%)<br>6/3739 (0.16%)<br>0/1963 (0%)<br>1/2161 (0.05%)<br>31/4786 (0.65%) | 7/5974 (0.12%)<br>2/3319 (0.06%)<br>0/1157 (0%)<br>0/1398 (0%)<br>0/3285 (0%) | 23 | (49), (50), (51), (52), (53), (54), (55), (56), (57), (58), (59), (60), (61), (63), (64), (65), (66), (67), (68), (69), (70), (71), (72) |
| Ashkenazi Jewish | p.L483P<br>p.N409S<br>p.T408M<br>p.E365K<br>p.R159W | 8/2689 (0.30%)<br>428/3117 (13.7%)<br>13/1935 (0.67%)<br>30/1935 (1.55%)<br>0/735 (0%) | 6/10175 (0.06%)<br>382/6795 (5.62%)<br>1/1000 (0.01%)<br>7/1000 (0.07%)<br>0/622 (0%) | 9 | (73), (74), (75), (76), (77), (78), (79), (80), (81) |

* The reported studies use either the conventional nomenclature for *GBA1* alleles excluding the 39-residue signal peptide or refer to the processed protein that includes the 39-residue signal peptide following Human Genome Variation Society (HGVS) recommendation. Articles investigating Norwegian and Scandinavian cohorts are highlighted in gray.

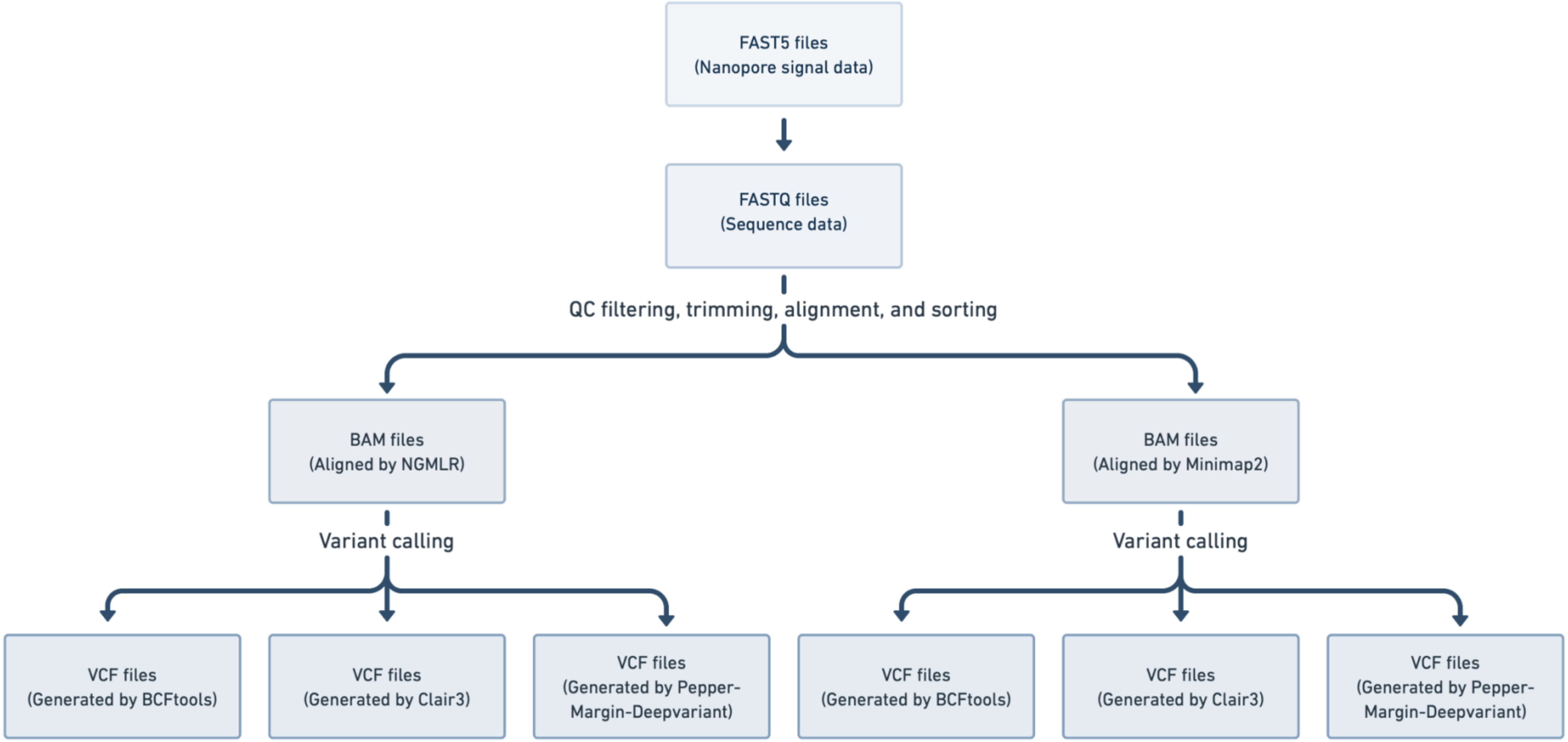


**Supplementary Figure 2.** Laboratory workflow data analysis pipeline of how the data was processed and which aligners (i.e. NGMLR and Minimap2) and variant callers (i.e. BCFtools, Clair3, Pepper-Margin-Deepvariant)

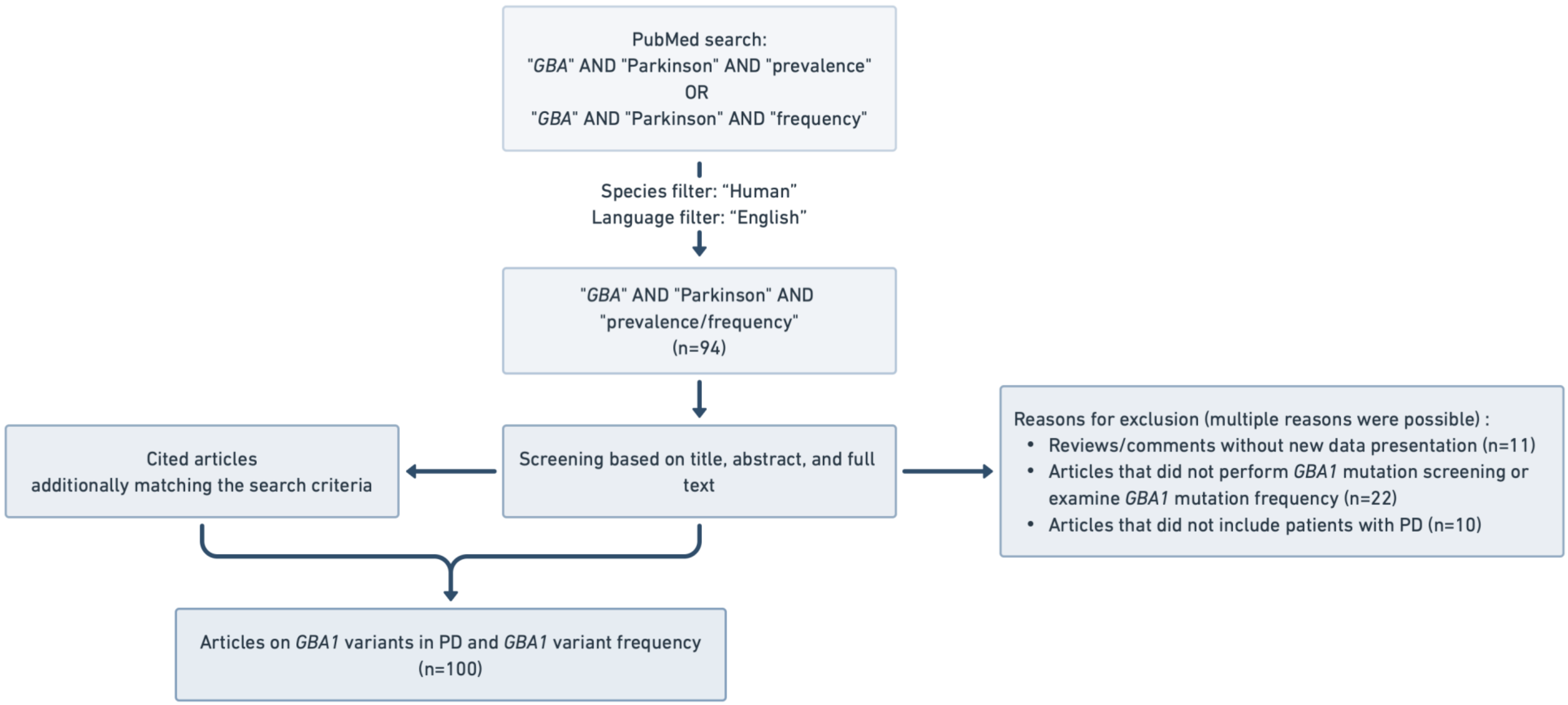


**Supplementary Figure 3.** Workflow of the literature search in PubMed. We searched for literature via PubMed that was published before August 4, 2022, using the search term "*GBA*" AND "Parkinson" AND "prevalence" OR "*GBA*" AND "Parkinson" AND "frequency", while setting the species filter to “Human” and the language filter to “English”, resulting in 94 articles. These were screened based on the title, abstract and full text, excluding all articles not directly screening for variants in the *GBA1* gene in patients with PD. Reasons for exclusion were reviews, or comments without new data (n=11), articles that did not perform *GBA1* variant screening or examine *GBA1* variant frequency in their study population (n=22), and articles that were not about PD or did not include patients with PD (n=10) (multiple reasons for exclusion were possible). In addition to the articles found via the search term, suitable articles that were referenced in this literature were also included in the overview. In the end, 100 articles on *GBA1* variant frequencies across populations were included in the overview

## References


1. Toft M, Pielsticker L, Ross OA, Aasly JO, Farrer MJ. Glucocerebrosidase gene mutations and Parkinson disease in the Norwegian population. Neurology. 2006;66(3):415-7.
2. Lunde KA, Chung J, Dalen I, Pedersen KF, Linder J, Domellof ME, et al. Association of glucocerebrosidase polymorphisms and mutations with dementia in incident Parkinson's disease. Alzheimers Dement. 2018;14(10):1293-301.
3. Berge-Seidl V, Pihlstrom L, Maple-Grodem J, Forsgren L, Linder J, Larsen JP, et al. The GBA variant E326K is associated with Parkinson's disease and explains a genome-wide association signal. Neurosci Lett. 2017;658:48-52.
4. Ran C, Brodin L, Gellhaar S, Westerlund M, Fardell C, Nissbrandt H, et al. Glucocerebrosidase variant T369M is not a risk factor for Parkinson's disease in Sweden. Neurosci Lett. 2022;784:136767.
5. Ran C, Brodin L, Forsgren L, Westerlund M, Ramezani M, Gellhaar S, et al. Strong association between glucocerebrosidase mutations and Parkinson's disease in Sweden. Neurobiol Aging. 2016;45:212 e5- e11.
6. Ylonen S, Siitonen A, Nalls MA, Ylikotila P, Autere J, Eerola-Rautio J, et al. Genetic risk factors in Finnish patients with Parkinson's disease. Parkinsonism Relat Disord. 2017;45:39-43.
7. Muldmaa M, Mencacci NE, Pittman A, Kadastik-Eerme L, Sikk K, Taba P, et al. Community-based genetic study of Parkinson's disease in Estonia. Acta Neurol Scand. 2021;143(1):89-95.
8. Neumann J, Bras J, Deas E, O'Sullivan SS, Parkkinen L, Lachmann RH, et al. Glucocerebrosidase mutations in clinical and pathologically proven Parkinson's disease. Brain. 2009;132(Pt 7):1783-94.
9. Winder-Rhodes SE, Evans JR, Ban M, Mason SL, Williams-Gray CH, Foltynie T, et al. Glucocerebrosidase mutations influence the natural history of Parkinson's disease in a community-based incident cohort. Brain. 2013;136(Pt 2):392-9.
10. Duran R, Mencacci NE, Angeli AV, Shoai M, Deas E, Houlden H, et al. The glucocerobrosidase E326K variant predisposes to Parkinson's disease, but does not cause Gaucher's disease. Mov Disord. 2013;28(2):232-6.
11. Olszewska DA, McCarthy A, Soto-Beasley AI, Walton RL, Magennis B, McLaughlin RL, et al. Association Between Glucocerebrosidase Mutations and Parkinson's Disease in Ireland. Front Neurol. 2020;11:527.
12. Crosiers D, Verstraeten A, Wauters E, Engelborghs S, Peeters K, Mattheijssens M, et al. Mutations in glucocerebrosidase are a major genetic risk factor for Parkinson's disease and increase susceptibility to dementia in a Flanders-Belgian cohort. Neurosci Lett. 2016;629:160-4.
13. den Heijer JM, Cullen VC, Quadri M, Schmitz A, Hilt DC, Lansbury P, et al. A Large-Scale Full GBA1 Gene Screening in Parkinson's Disease in the Netherlands. Mov Disord. 2020;35(9):1667-74.
14. Anheim M, Elbaz A, Lesage S, Durr A, Condroyer C, Viallet F, et al. Penetrance of Parkinson disease in glucocerebrosidase gene mutation carriers. Neurology. 2012;78(6):417-20.
15. Lesage S, Anheim M, Condroyer C, Pollak P, Durif F, Dupuits C, et al. Large-scale screening of the Gaucher's disease-related glucocerebrosidase gene in Europeans with Parkinson's disease. Hum Mol Genet. 2011;20(1):202-10.

16. Spataro N, Roca-Umbert A, Cervera-Carles L, Valles M, Anglada R, Pagonabarraga J, et al. Detection of genomic rearrangements from targeted resequencing data in Parkinson's disease patients. Mov Disord. 2017;32(1):165-9.

17. Seto-Salvia N, Pagonabarraga J, Houlden H, Pascual-Sedano B, Dols-Icardo O, Tucci A, et al. Glucocerebrosidase mutations confer a greater risk of dementia during Parkinson's disease course. Mov Disord. 2012;27(3):393-9.

18. Jesus S, Huertas I, Bernal-Bernal I, Bonilla-Toribio M, Caceres-Redondo MT, Vargas-Gonzalez L, et al. GBA Variants Influence Motor and Non-Motor Features of Parkinson's Disease. PLoS One. 2016;11(12):e0167749.

19. Bras J, Paisan-Ruiz C, Guerreiro R, Ribeiro MH, Morgadinho A, Januario C, et al. Complete screening for glucocerebrosidase mutations in Parkinson disease patients from Portugal. Neurobiol Aging. 2009;30(9):1515-7.

20. Petrucci S, Ginevrino M, Trezzi I, Monfrini E, Ricciardi L, Albanese A, et al. GBA-Related Parkinson's Disease: Dissection of Genotype-Phenotype Correlates in a Large Italian Cohort. Mov Disord. 2020;35(11):2106-11.

21. De Marco EV, Annesi G, Tarantino P, Rocca FE, Provenzano G, Civitelli D, et al. Glucocerebrosidase gene mutations are associated with Parkinson's disease in southern Italy. Mov Disord. 2008;23(3):460-3.

22. Asselta R, Rimoldi V, Siri C, Cilia R, Guella I, Tesei S, et al. Glucocerebrosidase mutations in primary parkinsonism. Parkinsonism Relat Disord. 2014;20(11):1215-20.

23. Cilia R, Tunesi S, Marotta G, Cereda E, Siri C, Tesei S, et al. Survival and dementia in GBA-associated Parkinson's disease: The mutation matters. Ann Neurol. 2016;80(5):662-73.

24. Straniero L, Asselta R, Bonvegna S, Rimoldi V, Melistaccio G, Solda G, et al. The SPID-GBA study: Sex distribution, Penetrance, Incidence, and Dementia in GBA-PD. Neurol Genet. 2020;6(6):e523.

25. Quadri M, Yang X, Cossu G, Olgiati S, Saddi VM, Breedveld GJ, et al. An exome study of Parkinson's disease in Sardinia, a Mediterranean genetic isolate. Neurogenetics. 2015;16(1):55-64.

26. Kalinderi K, Bostantjopoulou S, Paisan-Ruiz C, Katsarou Z, Hardy J, Fidani L. Complete screening for glucocerebrosidase mutations in Parkinson disease patients from Greece. Neurosci Lett. 2009;452(2):87-9.

27. Moraitou M, Hadjigeorgiou G, Monopolis I, Dardiotis E, Bozi M, Vassilatis D, et al. beta-Glucocerebrosidase gene mutations in two cohorts of Greek patients with sporadic Parkinson's disease. Mol Genet Metab. 2011;104(1-2):149-52.

28. Emekli I, Tepgec F, Samanci B, Toksoy G, Hasanogullari Kina G, Tufekcioglu Z, et al. Clinical and molecular genetic findings of hereditary Parkinson's patients from Turkey. Parkinsonism Relat Disord. 2021;93:35-9.

29. Kumar KR, Ramirez A, Gobel A, Kresojevic N, Svetel M, Lohmann K, et al. Glucocerebrosidase mutations in a Serbian Parkinson's disease population. Eur J Neurol. 2013;20(2):402-5.

30. Torok R, Zadori D, Torok N, Csility E, Vecsei L, Klivenyi P. An assessment of the frequency of mutations in the GBA and VPS35 genes in Hungarian patients with sporadic Parkinson's disease. Neurosci Lett. 2016;610:135-8.

31. Benitez BA, Davis AA, Jin SC, Ibanez L, Ortega-Cubero S, Pastor P, et al. Resequencing analysis of five Mendelian genes and the top genes from genome-wide association studies in Parkinson's Disease. Mol Neurodegener. 2016;11:29.

32. Noreau A, Riviere JB, Diab S, Dion PA, Panisset M, Soland V, et al. Glucocerebrosidase mutations in a French-Canadian Parkinson's disease cohort. Can J Neurol Sci. 2011;38(5):772-3.

33. Han F, Grimes DA, Li F, Wang T, Yu Z, Song N, et al. Mutations in the glucocerebrosidase gene are common in patients with Parkinson's disease from Eastern Canada. Int J Neurosci. 2016;126(5):415-21.

34. Sato C, Morgan A, Lang AE, Salehi-Rad S, Kawarai T, Meng Y, et al. Analysis of the glucocerebrosidase gene in Parkinson's disease. Mov Disord. 2005;20(3):367-70.

35. Gonzalez-Del Rincon Mde L, Monroy Jaramillo N, Suarez Martinez AI, Yescas Gomez P, Boll Woehrlen MC, Lopez Lopez M, et al. The L444P GBA mutation is associated with early-onset Parkinson's disease in Mexican Mestizos. Clin Genet. 2013;84(4):386-7.

36. Tipton PW, Soto-Beasley AI, Walton RL, Soler-Rangel S, Romero-Osorio O, Diaz C, et al. Prevalence of GBA p.K198E mutation in Colombian and Hispanic populations. Parkinsonism Relat Disord. 2020;73:16-8.

37. Velez-Pardo C, Lorenzo-Betancor O, Jimenez-Del-Rio M, Moreno S, Lopera F, Cornejo-Olivas M, et al. The distribution and risk effect of GBA variants in a large cohort of PD patients from Colombia and Peru. Parkinsonism Relat Disord. 2019;63:204-8.

38. Eblan MJ, Nguyen J, Ziegler SG, Lwin A, Hanson M, Gallardo M, et al. Glucocerebrosidase mutations are also found in subjects with early-onset parkinsonism from Venezuela. Mov Disord. 2006;21(2):282-3.

39. Dos Santos AV, Pestana CP, Diniz KR, Campos M, Abdalla-Carvalho CB, de Rosso AL, et al. Mutational analysis of GIGYF2, ATP13A2 and GBA genes in Brazilian patients with early-onset Parkinson's disease. Neurosci Lett. 2010;485(2):121-4.

40. Spitz M, Rozenberg R, Pereira Lda V, Reis Barbosa E. Association between Parkinson's disease and glucocerebrosidase mutations in Brazil. Parkinsonism Relat Disord. 2008;14(1):58-62.

41. Guimaraes Bde C, Pereira AC, Rodrigues Fda C, dos Santos AV, Campos M, Jr., dos Santos JM, et al. Glucocerebrosidase N370S and L444P mutations as risk factors for Parkinson's disease in Brazilian patients. Parkinsonism Relat Disord. 2012;18(5):688-9.

42. Socal MP, Bock H, Michelin-Tirelli K, Hilbig A, Saraiva-Pereira ML, Rieder CR, et al. Parkinson's disease and the heterozygous state for glucocerebrosidase mutations among Brazilians. Parkinsonism Relat Disord. 2009;15(1):76-8.

43. Barkhuizen M, Anderson DG, van der Westhuizen FH, Grobler AF. A molecular analysis of the GBA gene in Caucasian South Africans with Parkinson's disease. Mol Genet Genomic Med. 2017;5(2):147-56.

44. Mahungu AC, Anderson DG, Rossouw AC, van Coller R, Carr JA, Ross OA, et al. Screening of the glucocerebrosidase (GBA) gene in South Africans of African ancestry with Parkinson's disease. Neurobiol Aging. 2020;88:156 e11- e14.

45. Lesage S, Condroyer C, Hecham N, Anheim M, Belarbi S, Lohman E, et al. Mutations in the glucocerebrosidase gene confer a risk for Parkinson disease in North Africa. Neurology. 2011;76(3):301-3.

46. Nishioka K, Vilarino-Guell C, Cobb SA, Kachergus JM, Ross OA, Wider C, et al. Glucocerebrosidase mutations are not a common risk factor for Parkinson disease in North Africa. Neurosci Lett. 2010;477(2):57-60.

47. Emelyanov AK, Usenko TS, Tesson C, Senkevich KA, Nikolaev MA, Miliukhina IV, et al. Mutation analysis of Parkinson's disease genes in a Russian data set. Neurobiol Aging. 2018;71:267 e7- e10.

48. Emelyanov A, Boukina T, Yakimovskii A, Usenko T, Drosdova A, Zakharchuk A, et al. Glucocerebrosidase gene mutations are associated with Parkinson's disease in Russia. Mov Disord. 2012;27(1):158-9.

49. Mao XY, Burgunder JM, Zhang ZJ, An XK, Zhang JH, Yang Y, et al. Association between GBA L444P mutation and sporadic Parkinson's disease from Mainland China. Neurosci Lett. 2010;469(2):256-9.

50. Hu FY, Xi J, Guo J, Yu LH, Liu L, He XH, et al. Association of the glucocerebrosidase N370S allele with Parkinson's disease in two separate Chinese Han populations of mainland China. Eur J Neurol. 2010;17(12):1476-8.

51. Zhang X, Bao QQ, Zhuang XS, Gan SR, Zhao D, Liu Y, et al. Association of Common Variants in the Glucocerebrosidase Gene with High Susceptibility to Parkinson's Disease among Chinese. Chin J Physiol. 2012;55(6):398-404.

52. Guo JF, Li K, Yu RL, Sun QY, Wang L, Yao LY, et al. Polygenic determinants of Parkinson's disease in a Chinese population. Neurobiol Aging. 2015;36(4):1765 e1- e6.

53. Wang C, Cai Y, Gu Z, Ma J, Zheng Z, Tang BS, et al. Clinical profiles of Parkinson's disease associated with common leucine-rich repeat kinase 2 and glucocerebrosidase genetic variants in Chinese individuals. Neurobiol Aging. 2014;35(3):725 e1-6.

54. Ren J, Zhang R, Pan C, Xu J, Sun H, Hua P, et al. Prevalence and genotype-phenotype correlations of GBA-related Parkinson disease in a large Chinese cohort. Eur J Neurol. 2022;29(4):1017-24.

55. Yu Z, Wang T, Xu J, Wang W, Wang G, Chen C, et al. Mutations in the glucocerebrosidase gene are responsible for Chinese patients with Parkinson's disease. J Hum Genet. 2015;60(2):85-90.

56. Sun QY, Guo JF, Wang L, Yu RH, Zuo X, Yao LY, et al. Glucocerebrosidase gene L444P mutation is a risk factor for Parkinson's disease in Chinese population. Mov Disord. 2010;25(8):1005-11.

57. Wang Y, Liu L, Xiong J, Zhang X, Chen Z, Yu L, et al. Glucocerebrosidase L444P mutation confers genetic risk for Parkinson's disease in central China. Behav Brain Funct. 2012;8:57.

58. Tan EK, Tong J, Fook-Chong S, Yih Y, Wong MC, Pavanni R, et al. Glucocerebrosidase mutations and risk of Parkinson disease in Chinese patients. Arch Neurol. 2007;64(7):1056-8.

59. Li N, Wang L, Zhang J, Tan EK, Li J, Peng J, et al. Whole-exome sequencing in early-onset Parkinson's disease among ethnic Chinese. Neurobiol Aging. 2020;90:150 e5- e11.

60. Huang CL, Wu-Chou YH, Lai SC, Chang HC, Yeh TH, Weng YH, et al. Contribution of glucocerebrosidase mutation in a large cohort of sporadic Parkinson's disease in Taiwan. Eur J Neurol. 2011;18(10):1227-32.

61. Zhang Y, Sun QY, Zhao YW, Shu L, Guo JF, Xu Q, et al. Effect of GBA Mutations on Phenotype of Parkinson's Disease: A Study on Chinese Population and a Meta-Analysis. Parkinsons Dis. 2015;2015:916971.

62. Foo JN, Tan LC, Liany H, Koh TH, Irwan ID, Ng YY, et al. Analysis of non-synonymous-coding variants of Parkinson's disease-related pathogenic and susceptibility genes in East Asian populations. Hum Mol Genet. 2014;23(14):3891-7.

63. Ziegler SG, Eblan MJ, Gutti U, Hruska KS, Stubblefield BK, Goker-Alpan O, et al. Glucocerebrosidase mutations in Chinese subjects from Taiwan with sporadic Parkinson disease. Mol Genet Metab. 2007;91(2):195-200.

64. Gutti U, Fung HC, Hruska KS, Lamarca ME, Chen CM, Wu YR, et al. The need for appropriate genotyping strategies for glucocerebrosidase mutations in cohorts with Parkinson disease. Arch Neurol. 2008;65(6):850-1; author reply 1.

65. Wu YR, Chen CM, Chao CY, Ro LS, Lyu RK, Chang KH, et al. Glucocerebrosidase gene mutation is a risk factor for early onset of Parkinson disease among Taiwanese. J Neurol Neurosurg Psychiatry. 2007;78(9):977-9.

66. Choi JM, Kim WC, Lyoo CH, Kang SY, Lee PH, Baik JS, et al. Association of mutations in the glucocerebrosidase gene with Parkinson disease in a Korean population. Neurosci Lett. 2012;514(1):12-5.

67. Li Y, Sekine T, Funayama M, Li L, Yoshino H, Nishioka K, et al. Clinicogenetic study of GBA mutations in patients with familial Parkinson's disease. Neurobiol Aging. 2014;35(4):935 e3-8.

68. Mitsui J, Mizuta I, Toyoda A, Ashida R, Takahashi Y, Goto J, et al. Mutations for Gaucher disease confer high susceptibility to Parkinson disease. Arch Neurol. 2009;66(5):571-6.

69. Pulkes T, Choubtum L, Chitphuk S, Thakkinstian A, Pongpakdee S, Kulkantrakorn K, et al. Glucocerebrosidase mutations in Thai patients with Parkinson's disease. Parkinsonism Relat Disord. 2014;20(9):986-91.

70. Yadav R, Kapoor S, Madhukar M, Naduthota RM, Kumar A, Pal PK. Genetic analysis of the glucocerebrosidase gene in South Indian patients with Parkinson's disease. Neurol India. 2018;66(6):1649-54.

71. Biswas A, Sadhukhan D, Biswas A, Das SK, Banerjee TK, Bal PS, et al. Identification of GBA mutations among neurodegenerative disease patients from eastern India. Neurosci Lett. 2021;751:135816.

72. Halder T, Raj J, Pandey S, Kumar A, Kawale S, Chaudhary S, et al. Screening of Genetic Mutations in GBA1, GIGYF2 and VPS35 in Parkinson Disease Patients from India. Journal of Genetic Disorders & Genetic Reports. 2016.

73. Goldstein O, Gana-Weisz M, Cohen-Avinoam D, Shiner T, Thaler A, Cedarbaum JM, et al. Revisiting the non-Gaucher-GBA-E326K carrier state: Is it sufficient to increase Parkinson's disease risk? Mol Genet Metab. 2019;128(4):470-5.

74. Gan-Or Z, Giladi N, Rozovski U, Shifrin C, Rosner S, Gurevich T, et al. Genotype-phenotype correlations between GBA mutations and Parkinson disease risk and onset. Neurology. 2008;70(24):2277-83.

75. Gan-Or Z, Amshalom I, Kilarski LL, Bar-Shira A, Gana-Weisz M, Mirelman A, et al. Differential effects of severe vs mild GBA mutations on Parkinson disease. Neurology. 2015;84(9):880-7.

76. Dagan E, Schlesinger I, Ayoub M, Mory A, Nassar M, Kurolap A, et al. The contribution of Niemann-Pick SMPD1 mutations to Parkinson disease in Ashkenazi Jews. Parkinsonism Relat Disord. 2015;21(9):1067-71.

77. Liu X, Cheng R, Verbitsky M, Kisselev S, Browne A, Mejia-Sanatana H, et al. Genome-wide association study identifies candidate genes for Parkinson's disease in an Ashkenazi Jewish population. BMC Med Genet. 2011;12:104.

78. Ruskey JA, Greenbaum L, Ronciere L, Alam A, Spiegelman D, Liong C, et al. Increased yield of full GBA sequencing in Ashkenazi Jews with Parkinson's disease. Eur J Med Genet. 2019;62(1):65-9.

79. Clark LN, Nicolai A, Afridi S, Harris J, Mejia-Santana H, Strug L, et al. Pilot association study of the beta-glucocerebrosidase N370S allele and Parkinson's disease in subjects of Jewish ethnicity. Mov Disord. 2005;20(1):100-3.

80. Aharon-Peretz J, Rosenbaum H, Gershoni-Baruch R. Mutations in the glucocerebrosidase gene and Parkinson's disease in Ashkenazi Jews. N Engl J Med. 2004;351(19):1972-7.

81. Aharon-Peretz J, Badarny S, Rosenbaum H, Gershoni-Baruch R. Mutations in the glucocerebrosidase gene and Parkinson disease: phenotype-genotype correlation. Neurology. 2005;65(9):1460-1.

82. Gan-Or Z, Bar-Shira A, Mirelman A, Gurevich T, Kedmi M, Giladi N, et al. LRRK2 and GBA mutations differentially affect the initial presentation of Parkinson disease. Neurogenetics. 2010;11(1):121-5.

83. Alcalay RN, Levy OA, Waters CC, Fahn S, Ford B, Kuo SH, et al. Glucocerebrosidase activity in Parkinson's disease with and without GBA mutations. Brain. 2015;138(Pt 9):2648-58.

84. Clark LN, Ross BM, Wang Y, Mejia-Santana H, Harris J, Louis ED, et al. Mutations in the glucocerebrosidase gene are associated with early-onset Parkinson disease. Neurology. 2007;69(12):1270-7.

85. Alcalay RN, Caccappolo E, Mejia-Santana H, Tang MX, Rosado L, Ross BM, et al. Frequency of known mutations in early-onset Parkinson disease: implication for genetic counseling: the consortium on risk for early onset Parkinson disease study. Arch Neurol. 2010;67(9):1116-22.

86. Goker-Alpan O, Giasson BI, Eblan MJ, Nguyen J, Hurtig HI, Lee VM, et al. Glucocerebrosidase mutations are an important risk factor for Lewy body disorders. Neurology. 2006;67(5):908-10.

87. Sidransky E, Nalls MA, Aasly JO, Aharon-Peretz J, Annesi G, Barbosa ER, et al. Multicenter analysis of glucocerebrosidase mutations in Parkinson's disease. N Engl J Med. 2009;361(17):1651-61.

88. Mata IF, Leverenz JB, Weintraub D, Trojanowski JQ, Chen-Plotkin A, Van Deerlin VM, et al. GBA Variants are associated with a distinct pattern of cognitive deficits in Parkinson's disease. Mov Disord. 2016;31(1):95-102.

89. Nichols WC, Pankratz N, Marek DK, Pauciulo MW, Elsaesser VE, Halter CA, et al. Mutations in GBA are associated with familial Parkinson disease susceptibility and age at onset. Neurology. 2009;72(4):310-6.

90. Liu G, Boot B, Locascio JJ, Jansen IE, Winder-Rhodes S, Eberly S, et al. Specifically neuropathic Gaucher's mutations accelerate cognitive decline in Parkinson's. Ann Neurol. 2016;80(5):674-85.

91. Stoker TB, Camacho M, Winder-Rhodes S, Liu G, Scherzer CR, Foltynie T, et al. Impact of GBA1 variants on long-term clinical progression and mortality in incident Parkinson's disease. J Neurol Neurosurg Psychiatry. 2020;91(7):695-702.

92. Gorostidi A, Marti-Masso JF, Bergareche A, Rodriguez-Oroz MC, Lopez de Munain A, Ruiz-Martinez J. Genetic Mutation Analysis of Parkinson's Disease Patients Using Multigene Next-Generation Sequencing Panels. Mol Diagn Ther. 2016;20(5):481-91.

93. Mata IF, Samii A, Schneer SH, Roberts JW, Griffith A, Leis BC, et al. Glucocerebrosidase gene mutations: a risk factor for Lewy body disorders. Arch Neurol. 2008;65(3):379-82.

94. Lwin A, Orvisky E, Goker-Alpan O, LaMarca ME, Sidransky E. Glucocerebrosidase mutations in subjects with parkinsonism. Mol Genet Metab. 2004;81(1):70-3.

95. Malek N, Weil RS, Bresner C, Lawton MA, Grosset KA, Tan M, et al. Features of GBA-associated Parkinson's disease at presentation in the UK Tracking Parkinson's study. J Neurol Neurosurg Psychiatry. 2018;89(7):702-9.

96. Eblan MJ, Walker JM, Sidransky E. The glucocerebrosidase gene and Parkinson's disease in Ashkenazi Jews. N Engl J Med. 2005;352(7):728-31; author reply -31.

97. Barber TR, Lawton M, Rolinski M, Evetts S, Baig F, Ruffmann C, et al. Prodromal Parkinsonism and Neurodegenerative Risk Stratification in REM Sleep Behavior Disorder. Sleep. 2017;40(8).

98. Malec-Litwinowicz M, Rudzinska M, Szubiga M, Michalski M, Tomaszewski T, Szczudlik A. Cognitive impairment in carriers of glucocerebrosidase gene mutation in Parkinson disease patients. Neurol Neurochir Pol. 2014;48(4):258-61.

99. McNeill A, Duran R, Hughes DA, Mehta A, Schapira AH. A clinical and family history study of Parkinson's disease in heterozygous glucocerebrosidase mutation carriers. J Neurol Neurosurg Psychiatry. 2012;83(8):853-4.

100. Graham OEE, Pitcher TL, Liau Y, Miller AL, Dalrymple-Alford JC, Anderson TJ, et al. Nanopore sequencing of the glucocerebrosidase (GBA) gene in a New Zealand Parkinson's disease cohort. Parkinsonism Relat Disord. 2020;70:36-41.
